# Evaluation of Personas and Daily Activity Maps to Inform Clinical Care and Socially Assistive Robot Training and Design for Persons With Dementia: Mixed Methods Study

**DOI:** 10.2196/85651

**Published:** 2026-10-28

**Authors:** Adeline Nyamathi, Mahkameh Rasouli, Cheonkam Jeong, Sabine Brunswicker, Elena Agapie, Jocelyn Ludlow, Riyam Jawad, Chet Khay, Audrey Lu, Jessica Ding Liao, Saba Azizabadi Farahani, Jung-Ah Lee, Nikil Dutt, Amir Rahmani, Erik Krogh

**Affiliations:** 1 Sue & Bill Gross School of Nursing, University of California Irvine, 854 Health Sciences Rd, Irvine, CA, 92697, United States, 1 949-824-8932; 2 Donald Bren School of Information and Computer Sciences, University of California Irvine, Irvine, CA, United States; 3 Research Center for Digital Innovation, Purdue University, West Lafayette, IN, United States; 4 NaviGAIT, Irvine, CA, United States; 5 Amore Senior Living, Laguna Niguel, CA, United States; 6 Department of Electrical Engineering and Computer Science, Henry Samueli School of Engineering, University of California Irvine, Irvine, CA, United States; 7 Smart Forward, Rancho Palos Verdes, CA, United States

**Keywords:** dementia, persona, daily activity map, human-robot interaction, socially assistive robot

## Abstract

**Background:**

Over 55 million people worldwide are living with dementia, resulting in significant caregiving demands and caregiver burden. Socially assistive robots (SARs) are increasingly being explored to reduce caregiving workload and improve the quality of life for persons with dementia; however, their effectiveness depends on person-centered design grounded in a comprehensive understanding of the target population. Personas and their corresponding daily activity maps (DAMs) are user-centered design tools that provide a holistic understanding of the personal, medical, emotional, and life trajectory of persons with dementia, enabling SARs to engage in contextually relevant and empathetic interactions. Despite their potential, these tools have not been formally evaluated for dementia care and digital health technology development.

**Objective:**

This mixed methods study aimed to explore the perspectives of professional and family caregivers, health care providers, and robot designers on the quality and use of personas and DAMs in clinical practice and digital health design.

**Methods:**

Using purposive sampling, 15 stakeholders involved in dementia care or digital health design were interviewed via Zoom (Zoom Communications, Inc) between March and April 2025 to evaluate a subset of persona-DAM pairs using a modified 12-item version of the Persona Perception Scale (PPS), adapted for dementia care contexts, across 5 domains: Credibility, Completeness, Clarity, Willingness to Use, and Empathy. Participants subsequently provided qualitative perspectives through semistructured interviews, reflecting on their ratings and offering suggestions for improvement.

**Results:**

Fifteen participants with a median of 9 (IQR 4-15) years of experience in dementia care evaluated 9 persona-DAM pairs. Overall evaluation-level median PPS scores were 4.79 (IQR 4.45, 4.91) for personas and 4.76 (IQR 4.45, 4.94) for DAMs. For both personas and DAMs, the highest-rated domains were Clarity, Credibility, and Willingness to Use (all median 5.00), suggesting stakeholders perceived the tools favorably and were willing to use them. Empathy scores (personas: median 4.67, IQR 4.25-5; DAMs: median 4.83, IQR 4.33-5) highlighted the need for greater cultural, ethnic, and religious representation to foster culturally sensitive interventions. Completeness was the lowest-scoring domain (personas: median 4.50, IQR 3.94-5; DAMs: median 4.38, IQR 4-5), representing the most quantitative improvement suggestions. Qualitative analysis revealed 3 major themes: (1) improving human-robot interaction training through greater knowledge of the personal context of persons with dementia; (2) comprehensive assessment of clinical history and functional capacities as essential for the provision of safe and effective interaction; and (3) perceived design utility: translating person-centered knowledge into actionable tools.

**Conclusions:**

This study provides early-stage exploratory evidence that personas and DAMs are perceived as clinically and technically relevant tools for dementia care, complementing existing clinical documentation through a holistic, person-centered view, and serving as human-centered design tools for developing personalized digital health solutions.

## Introduction

Dementia is a progressive neurodegenerative disorder currently estimated to affect over 55 million individuals worldwide [1], with projections indicating this number will nearly triple to 153 million by 2050 [2]. In the United States alone, approximately 7.2 million individuals are living with dementia [3]. With prevalence increasing as populations age [4], the impact on family and professional caregiving has become substantial, contributing to high levels of caregiver burden, burnout, and psychological distress [5]. As cognitive function declines, persons with dementia frequently exhibit behavioral and psychological symptoms of dementia, including agitation, wandering, and confusion [6]. These behaviors increase fall risk by at least twice compared to persons without cognitive decline [7], contributing to unnecessary hospitalization and preventable mortality [8].

With advancing technology and user-centered design approaches, Socially Assistive Robots (SARs) have emerged as a promising means of supporting caregivers in anticipating and responding to the needs of persons with dementia through social interaction [9-11]. Over the past 2 decades, a growing body of research has examined the application of SARs in dementia care across a range of contexts, including hospitals, home care settings, and residential dementia care facilities. Among the most extensively studied platforms is PARO (Personal Assistive Robot, PARO Robots, Inc), a robotic therapeutic seal developed in Japan, which has been evaluated in multiple randomized and quasi-experimental trials [12-15]. Moyle et al demonstrated that interactions with PARO were associated with reductions in agitation, improvements in mood, and increased social engagement among residents with dementia in aged care facilities [12]. Similarly, Wada and Shibata [16] reported physiological and psychological benefits of prolonged PARO use in care home settings, including reductions in stress-related hormones. Beyond PARO, humanoid robots such as NAO (SoftBank Robotics Corp) and Pepper (SoftBank Robotics Corp) have been piloted in dementia care environments to facilitate cognitive exercises, reminiscence activities, and caregiver support functions [17-19]. A further notable platform is MARIO (Managing Active and healthy aging with use of caring service Obots; Robosoft Technologies), developed specifically to support the psychosocial wellbeing of persons with dementia. D’Onofrio et al [20] reported that MARIO (Robosoft Technologies) facilitated social engagement, reduced feelings of loneliness, and supported reminiscence and cognitively stimulating activities among community care and home care residents with mild-to-moderate dementia. A scoping review by Abdi et al [21] of 107 studies similarly concluded that SARs hold promise for improving emotional well-being and reducing social isolation among care home residents, and a systematic review by Bemelmans et al [22] examining 21 studies found that SARs produced predominantly positive effects on mood, social behavior, and quality of life in older adults.

Although many SARs use AI to adapt interventions to the behavior of individual users over time, such as preferences, patterns of engagement, and daily activities, the primary personalization at the design stage relies on relatively brief profiles of the users [21,23]. These profiles lack integration of patients’ life histories, cultural backgrounds, personal relationships, and individual behavioral triggers, information that is essential for meaningful human-robot interaction [12,24]. This gap is particularly critical in dementia care, where progressive cognitive impairment limits the ability of persons with dementia to self-report such information accurately, fundamentally limiting the capacity of SARs to deliver truly person-centered interactions and to equip caregivers with the contextual knowledge needed to manage the psychobehavioral manifestations of dementia.

Life story work, defined as the structured collection and use of biographical information to guide care planning, has been associated with improvements in person-centered care (PCC) delivery, caregiver empathy, and reduced agitation in persons with dementia [25]. McKeown et al [25] found that life story approaches helped care staff understand the meaning behind challenging behaviors, reframing them as expressions of unmet needs rooted in personal history rather than symptoms to be managed [25]. If robots are to provide personalized interactions, they must therefore be informed by the individual’s life trajectory, their daily routines, cultural background, significant relationships, and preferred activities [26]. Personas and their associated daily activity maps (DAMs), as examined in this study, represent a structured approach for translating such life trajectory data into actionable design inputs for both SAR developers and caregivers. Specifically, personas can provide caregivers and SAR designers with a more nuanced understanding of the diverse needs and behaviors of persons with dementia, support the development of more intuitive and user-friendly SARs [27], facilitate empathy and understanding of diverse patient populations [28], support training of new caregivers [29], and assist in designing supportive care environments [30,31]. The DAM further identifies potential triggers for agitation and fall risk within a representative patient’s daily routine, enabling the proactive design of preventive and calming strategies [10].

However, the utility of personas and DAMs depends critically on their validity and perceived relevance to real-world clinical practice. If personas are not grounded in robust, empirical research, they risk reinforcing stereotypes [32], oversimplifying complex patient presentations, and failing to capture the evolving nature of dementia over time [28]. While personas and DAMs have been applied in various health design contexts [33-40], their specific utility and validity for SAR design in dementia care and the perspectives of the diverse stakeholders who would use them have not been systematically evaluated. In dementia care specifically, acceptance of technology is further complicated by the cognitive limitations of persons with dementia, which places a disproportionate burden of evaluation and acceptance judgment on professional and family caregivers acting on behalf of direct care recipients [41,42]. Addressing this gap requires a multidisciplinary approach combining insights from nursing, medicine, gerontology, psychology, robotics, and human-computer interaction (HCI), necessitating close collaboration with stakeholders across the care continuum.

The purpose of this mixed methods study was to investigate the perspectives of stakeholders involved in the care of persons with dementia, including professional caregivers (dementia nurse scientists, dementia registered nurses, physicians, psychiatrists, and nurse aides), family caregivers, and health-tech and robot designers, on the application of personas and their DAMs in designing digital health solutions for persons living with dementia. The research questions were as follows:

1. How well do personas and their DAMs of representative persons with dementia score on a modified 12-item version of the Persona Perception Scale (PPS) [43], as a quantitative measure of persona quality?
2. What are the perspectives of dementia stakeholders on the strengths and areas for improvement of these personas and DAMs for designing technology to better understand patient needs and behavioral triggers?

## Methods

### Design

Our parent mixed methods study was primarily designed to support the development of a conversational AI agent using large language models (LLMs) that will enable a SAR to provide empathetic conversation, particularly useful when a person with dementia is seemingly agitated [44]. In this study, we focused on developing personas and DAMs to promote an understanding of representative persons with dementia and evaluated and improved them by engaging key stakeholder perspectives. The enhanced personas and DAMs guide the design process for the dementia-informed conversational AI in the parent study [44]. A full description of the personas and their DAMs has been presented by Krogh et al [45].

### Conceptual Framework: HCI

The conceptual framework for this study is grounded in HCI design principles [46], specifically focusing on the effectiveness and user experience of digital systems. Two core principles of HCI are particularly salient for this study: user-centered design (UCD) [47] and Affective Computing [48]. UCD posits that the needs and lived experiences of the end user must drive every stage of the design process [47]. In the context of dementia care, this means that digital health solutions, including SARs and conversational AI agents, must be informed by a deep understanding of the life story work and medical records of persons with dementia. Personas and DAMs operationalize this principle by translating complex, individual-level data into accessible design artifacts that keep the person with dementia, rather than the technology, at the center of the design process. Affective Computing focuses on the capacity of systems to recognize, interpret, and respond to human emotional states [49]. For persons with dementia, who may experience heightened states of agitation, anxiety, or emotional distress, the ability of a SAR or conversational AI to respond empathetically is critical to its acceptability and effectiveness. Personas and DAMs support this by helping developers understand the behavioral, emotional, and sociocultural dimensions of persons with dementia, fostering empathy in designers who may have limited direct exposure to this population.

These 2 principles are directly reflected in the 5 constructs of the PPS [43] used in this study (1) “Credibility,” or the perception that personas and their DAMs are trustworthy and the likelihood that they will be engaged by end users [32]; (2) “Completeness,” which relates to a holistic collection of information about a person that would be helpful to clinicians in terms of decision-making as well as to scientists and digital health developers; (3) “Clarity,” which focuses on how clearly the information is presented in the personas and their DAMs in terms of understandability, format, and visibility; (4) “Empathy,” which relates to understanding or feeling a connection to a person on a personal level [50]; and (5) “Willingness to Use,” where if all other characteristics are strong, the willingness of the end user to engage with the tools would be optimal. Credibility, Completeness, and Clarity align with UCD by ensuring that personas and DAMs are trustworthy, holistically informative, and clearly interpretable across multidisciplinary stakeholder groups. Empathy directly reflects the Affective Computing principle, assessing whether the personas and DAMs foster an emotional connection to and understanding of the person with dementia. Willingness to Use serves as an integrative outcome measure, indicating whether the tools are perceived as sufficiently user-centered and empathy-inducing to be adopted in clinical and design practice.

### Development of Personas and DAMs

A Design-Use research team, consisting of experts from nursing, medicine, psychology, computer science, engineering, HCI, and business, was formed to provide expert guidance on dementia-care contexts and to develop personas and their associated DAMs. All team members had prior experience with robotics applications in dementia care. Drawing on the members’ clinical or caregiving experience, expertise in designing digital solutions for persons with dementia, and representative schemas of individuals who had resided in team members’ facilities, the team constructed 9 personas (eg, “Norman,” “Denise”) and corresponding DAMs over a 3 months through a series of iterative meetings (Multimedia Appendix 1). The persona names are pseudonymous and are assigned to fictional composite profiles representing persons with dementia, and they do not correspond to any actual study participants. Miro  (RealtimeBoard, Inc) [51] served as the collaborative platform for persona creation, while Canva (Canva, Inc) [52] was used to create the DAMs. In parallel, the team codeveloped a semistructured interview guide to facilitate the one-on-one qualitative interviews.

### Modified PPS

The PPS is a validated instrument originally comprising 31 items designed to evaluate the quality of persona constructs [43]. The original scale was developed and validated in the context of HCI and technology design; however, it has not been validated for use in dementia-care contexts. Therefore, the Design-Use research team conducted an extensive expert review of the PPS items and engaged in iterative discussions aimed at adapting the instrument for the dementia care context, retaining applicable items, removing unrelated content, thus avoiding a repetitive and lengthy scale to reduce participant fatigue. Through this process, the original 31-item scale was adapted to a concise 12-item version with minor wording modifications to enhance clinical relevance for caregivers of persons with dementia. Each item is scored on a 5-point Likert scale across 5 characteristics: Credibility, Completeness, Clarity, Empathy, and Willingness to Use. The same modified items were subsequently applied to the evaluation of the DAMs. The modified PPS for both persona and DAM evaluation has been presented as Multimedia Appendix 2.

### Sample and Setting

Participants were recruited through purposive referral by the research team, targeting individuals with documented expertise in dementia in the relevant stakeholder fields, including family and professional caregiving, nursing practice and research, medicine, psychiatry, and robot development and user interface (UI) and user experience (UX) design, to represent a multidisciplinary array of experts well qualified to provide insights on the existing personas and their DAMs and to make recommendations for improvement. Inclusion criteria required participants to be aged 18 or older and to have at least 6 months of expertise in the care of, or work related to improving the care of, persons with dementia. A target sample size of 15 was determined a priori as adequate for an exploratory, formative study of this type, with the final sample size contingent on data saturation. Of the 19 individuals invited via email, 15 accepted and were interviewed. The 4 individuals who did not respond were not followed up, in keeping with the principles of voluntary participation; reasons for nonresponse are unknown. Participants were interviewed individually via Zoom (Zoom Communications, Inc) [53] over sessions of 60‐75 minutes. Data were collected between March 17 and April 22, 2025.

### Procedure

This assessment was conducted by a trained researcher (AN) and a graduate student (MR), using a structured quantitative scale, the modified 12-item version of the PPS [43], to assess the usability of personas and their DAMs to help in designing successful human-robot interaction, alongside one-on-one interviews, explaining areas of further improvement for any score less than “Agree” (a score of 4 on the modified PPS Likert scale). The COREQ (Criteria for Reporting Qualitative Research) [54] was used to ensure a consistent process throughout the study. The COREQ checklist has been provided in Checklist 1.

Prior to the interview session, the 9 personas and their DAMs were sent to all interested stakeholders. Stakeholders were asked to become familiar with and select up to 3 personas and their DAMs for discussion, based on their familiarity and relatability with the persona on a personal level. This decision was made by the Design-Use team to avoid excessive respondent burden.

At the beginning of the interview session via Zoom (Zoom Communications, Inc) [53], a brief demographic questionnaire was collected, and the purpose of the meeting was reiterated, focused on improving the quality and detail of information provided in the personas and their DAMs. After each persona and its corresponding DAM were re-reviewed to refresh the stakeholder’s memory, the interviewer assessed each item on the modified PPS Likert scale of 1 (strongly disagree) to 5 (strongly agree). When scores were below 4 (“Agree”), the interviewer explored areas where the personas and their DAMs could be improved. The first author (AN) used the interview guide (Multimedia Appendix 3) to lead the individual interviews.

The rationale for selecting individual interviews as the qualitative component arose primarily from the mixed methods design of the study. First, each stakeholder evaluated a different, self-selected subset of persona-DAM pairs, chosen on the basis of personal familiarity and relatability, so there was no common set of materials on which a group discussion could be anchored. Second, the qualitative component was directly linked to each participant’s own quantitative ratings, based on which the interviewer probed further to collect qualitative insights into the reasons for the rating and desired improvements. Thus, the quantitative data determined the direction of the qualitative inquiry, reflecting the integration of the quantitative and qualitative components at the level of individual participants and items.

### Data Analysis

Sample characteristics, including race and ethnicity, area of expertise, and years of experience, were summarized using frequencies and percentages for categorical variables, and median (IQR) for the continuous variable. Consistent with the PPS psychometric guide [43], total scores were calculated for each of the 5 domains, Credibility, Completeness, Clarity, Willingness to Use, and Empathy, by averaging the item scores associated with each domain, for both persona and DAM evaluations. To facilitate interpretation and comparison, an overall PPS score was computed by averaging the 5 domain-specific mean scores. While the overall PPS score is not part of the original validated instrument, it is reported here solely for descriptive and exploratory purposes. Given the ordinal nature of the data, the small sample size, and the negatively skewed distribution, all domain and overall scores are reported as median (IQR). Two descriptive analyses were performed using different units of analysis. First, the evaluation-level analysis included all 36 persona-DAM evaluations. To assess whether repeated evaluations of different persona-DAM pairs by the same stakeholder influenced the results, a participant-level analysis (n=15) was also conducted. For this analysis, ratings were first averaged within each participant across all persona-DAM pairs they evaluated, so that each participant contributed one score per domain. Missing item responses were excluded from the calculation of the corresponding domain mean. All analyses were performed using R (R Core Team; version  4.4.3).

Qualitative data analysis was led and overseen by the first author (AN) along with a graduate student (MR) trained in qualitative research. All interviews were audio-recorded with permission and transcribed using Otter.ai (Otter.ai, Inc) [55] within 5 days of the interview. Each recording was reviewed against the transcribed interview to ensure the recording was accurate. Minor revisions and typos were corrected. These transcriptions were deidentified and stored securely under encryption, and accessible only to the research team.

Following the completion of the transcription process, thematic analysis was conducted by 2 independent researchers (AN and MR), using a reflexive approach within the constructivist paradigm [56-58]. Consistent with the reflexive approach [56-58], the analysts’ backgrounds were engaged as interpretive resources. AN is a Distinguished Professor of Nursing with over 40 years of community-based research with vulnerable populations, including persons with dementia, and extensive experience in qualitative research. MR is a PhD candidate with research experience in digital health. AN and MR have both collaborated on studies of remote health monitoring and user experience research for persons with dementia. The analysts’ experiences informed the interpretation of stakeholders’ insights, and ongoing reflexive discussions were used to deepen and challenge the analysts’ understanding of the data. The analysis followed the 6-phase process outlined by Braun and Clarke [56]: (1) Familiarization with the data: both AN and MR independently engaged with all 15 transcripts and recorded initial impressions. (2) Generating initial codes: both analysts independently generated initial codes from the first 5 transcripts, then met in a reflexive meeting to discuss and compare their interpretations. This process was repeated for the remaining transcripts in batches of 5, with coding iteratively refined across the full 15 transcripts. Dataset adequacy was assessed based on information power. (3) Searching for themes: AN and MR independently grouped codes into candidate themes representing broader patterns of shared meaning. Candidate themes were subsequently discussed and refined in weekly reflexive meetings, with attention to the central organizing concept underlying each theme. (4) Reviewing themes: both analysts reviewed all candidate themes against the full dataset to ensure coherence and representativeness. (5) Defining and naming themes: both analysts collaboratively refined theme definitions and names to clearly articulate the central organizing concept represented by each theme and how the themes collectively addressed the broader research question. (6) Producing the final report: AN led manuscript writing, while MR reviewed all quoted data for accuracy and representativeness.

As is standard in qualitative analysis, trustworthiness of data collection and analysis is established through credibility, confirmability, transferability, and dependability [59]. Credibility was ensured by engaging a team of researchers, health care providers, and robot designers who held a common purpose to support digital health technology development for persons with dementia. Transferability was ensured by providing a detailed description of the data collection procedure and methods. In addition, all interviews were conducted privately, audio-recorded, and transcribed, with a senior coder overseeing the entire process. Dependability was ensured by describing the design and implementation of the study, while confirmability was ensured through ongoing discussions of the day-to-day descriptions of the lives of persons with dementia in dementia care facilities and understanding the culture and values of persons with dementia from the perspectives of their caregivers.

### Ethical Considerations

This study was approved by the Institutional Review Board (IRB) of the University of California, Irvine (IRB #5816). All study procedures were conducted in accordance with relevant ethical guidelines and regulations. Prior to participation, all individuals provided oral informed consent. At the beginning of the interview session, ongoing consent to participate was reassessed and orally confirmed. At the end of the interview, participants were offered an electronic gift (e-gift) card for their participation.

## Results

### Overview

Depending on the length of time needed to discuss each persona and their DAM, ranging from 15 to 60 minutes each, the average number of personas and DAMs reviewed per participant was 2.4 (SD 0.63), resulting in 36 total evaluations. The overwhelming majority of personas and DAMs were each reviewed by 4 different stakeholders, with the exception of Norman, who was interviewed by 3 stakeholders, and Billy, who was reviewed by 5 stakeholders.

### Sociodemographic Data

As reflected in Table 1, the 15 stakeholders included 11 females and 4 males. Their racial and ethnic distribution was as follows: Non-Hispanic White (n=7), African American (n=3), Asian (n=2), Middle Eastern (n=2), and Hispanic White (n=1). Among these stakeholders, the following disciplines were represented: practicing registered nurses (n=2); nurse scientists (n=3); facility nurse caregivers who were also involved in administrative duties (n=2); facility nonnurse caregivers (n=2); family caregivers (n=2); medical providers specializing in dementia care (1 geriatrician and 1 psychiatrist); and human-robot designers (n=2). Participants had a median of 9 (IQR 4-15) years of experience caring for or designing digital health solutions for persons with dementia.

**Table 1.** Stakeholder sociodemographic characteristics (N=15).

Characteristics	Values
Sex, n (%)	
Female	11 (73.33)
Male	4 (26.66)
Race, n (%)	
African American	3 (20.0)
Asian	2 (13.3)
Hispanic White	1 (6.7)
Middle Eastern	2 (13.3)
Non-Hispanic White	7 (46.7)
Job title, n (%)	
Practicing registered nurse	2 (13.3)
Nurse scientist or researcher	3 (20.0)
Facility nurse caregiver or administrator	2 (13.3)
Facility nonnurse caregiver	2 (13.3)
Family caregivers	2 (13.3)
Physician-geriatrician	1 (6.7)
Psychiatrist	1 (6.7)
Human-robot designer or robot engineer	2 (13.3)
Experience (years), median (IQR)	9.00 (4-15)

### Quantitative Findings

As presented in Table 2, the evaluation-level analysis (N=36) showed generally high ratings for the personas across all modified PPS domains. The overall persona evaluation score had a median of 4.79 (IQR 4.45, 4.91). Analysis of the subscales revealed that the highest-rated domains were Willingness to Use (median 5.00, IQR 4.67-5.00), Clarity (median 5.00, IQR 4.67-5.00), and Credibility (median 5.00, IQR 4.62-5.00); all receiving the maximum median score on the scale, indicating consistent agreement among participants. The narrow IQRs across these domains, combined with median scores at the ceiling, reflect a negatively skewed distribution in which the majority of ratings were clustered at the high end of the scale. Empathy received a median score of 4.67 (IQR 4.25-5.00). Completeness received the lowest median score among the subscales (median 4.50, IQR 3.94-5.00); its wider IQR indicates greater variability in participant perceptions regarding the comprehensiveness of the personas’ information. The participant-level analysis (n=15), in which ratings were averaged across all persona-DAM pairs evaluated by each participant, showed a similar pattern in the magnitude and ordering of scores. Clarity received the highest median score (median 5.00, IQR 4.56-5.00), followed by Willingness to Use (median 4.89, IQR 4.67-5.00) and Credibility (median 4.83, IQR 4.61-5.00). Empathy (median 4.56, IQR 4.28-4.86) and Completeness (median 4.42, IQR 4.17-4.69) remained the comparatively lower-rated domains. The similarity between the 2 evaluation-level and participant-level descriptive analyses suggests that the overall pattern of ratings was not substantially influenced by participants who contributed multiple evaluations.

**Table 2.** Modified PPS^a^ descriptive statistics for personas and DAMs^b^.

Modified PPS	Evaluation-level^c^ (N=36)	Participant-level^d^ (N=15)
Personas, median (IQR)		
Overall persona evaluation score^e^	4.79 (4.45-4.91)	4.76 (4.46-4.87)
Completeness total score	4.5 (3.94-5)	4.42 (4.17-4.69)
Willingness to use total score	5 (4.67-5)	4.89 (4.67-5)
Credibility total score	5 (4.62-5)	4.83 (4.61-5)
Clarity total score	5 (4.67-5)	5 (4.56-5)
Empathy total score	4.67 (4.25-5)	4.56 (4.28-4.86)
DAMs, median (IQR)		
Overall DAM evaluation score^e^	4.76 (4.45-4.94)	4.83 (4.41-4.93)
Completeness total score	4.38 (4-5)	4.5 (3.96-4.73)
Willingness to use total score	5 (5-5)	5 (4.86-5)
Credibility total score	5 (4.67-5)	5 (4.56-5)
Clarity total score	5 (4.25-5)	4.78 (4.17-5)
Empathy total score	4.83 (4.33-5)	4.67 (4.22-5)

a PPS: Persona Perception Scale.

b DAM: daily activity map.

c Evaluation-level (N=36) used all persona-DAM evaluations as the unit of analysis.

d Participant-level analysis (n=15) used participants as the unit of analysis; for participants who evaluated more than one persona-DAM pair, ratings were averaged within participant across all evaluated pairs (mean 2.4; SD 0.63 pairs per participant; 36 evaluations total), so that each participant contributed one score per domain.

e The Overall score is an author-created composite, calculated as the mean of the 5 domain scores; it is not part of the validated PPS and is reported for descriptive purposes only.

A similar pattern was observed for the DAMs. At the evaluation level (N=36), the overall DAM evaluation score had a median of 4.76 (IQR 4.45-4.94). Consistent with the persona evaluation, Willingness to Use (median 5.00, IQR 5.00-5.00), Credibility (median 5.00, IQR 4.67-5.00), and Clarity (median 5.00, IQR 4.25-5.00) were the highest-rated domains. Notably, the IQR for DAM Willingness to Use was [5.00, 5.00], indicating a complete ceiling effect across all evaluations. Empathy also received a high rating (median 4.83, IQR 4.33-5.00), though slightly below the 3 ceiling-level domains. As with the personas, Completeness was the lowest-rated domain for the DAMs (median 4.38, IQR 4.00-5.00), with the widest IQR among all DAM subscales, reflecting the greatest variability in participant responses. The participant-level analysis (n=15) showed a materially similar pattern, with Willingness to Use (median 5.00, IQR 4.86-5.00) and Credibility (median 5.00, IQR 4.56-5.00) receiving the highest ratings, followed by Clarity (median 4.78, IQR 4.17-5.00) and Empathy (median 4.67, IQR 4.22-5.00). Completeness remained the lowest-rated domain (median 4.50, IQR 3.96-4.73).

These descriptive findings are exploratory and provide insight into participants’ perceptions of personas and DAMs as design tools intended to inform future SAR development for dementia care. These findings do not provide evidence of effectiveness or clinical benefit, as such conclusions require future clinical implementation and empirical evaluation.

### Qualitative Findings

The qualitative findings below highlight in detail the suggestions of the stakeholders in improving the knowledge base for future design of the personas and their DAMs. The analysis of the one-on-one interview data revealed 3 major themes: (1) improving human-robot interaction training through greater knowledge of the personal context of persons with dementia, (2) comprehensive assessment of clinical history and functional capacities as essential for the provision of safe and effective interaction for persons with dementia, and (3) perceived design utility: translating person-centered knowledge into actionable tools.

#### Improving Human-Robot Interaction Training Through Greater Knowledge of the Personal Context of Persons With Dementia

This theme captures the expressed need for the personas and DAMs to contain more contextual personal information, including both biographical history and emotional and sociobehavioral details. The theme also reflects the understanding that effective care requires knowledge beyond medical conditions, encompassing the full lived experience and personal narrative of individuals to create more useful and humanistic AI tools. This knowledge would be gained by gathering information from social networks surrounding the patient, including health care providers, family, and friends.

##### Need for a Better Understanding of the Life of the Person Before Living With Dementia

While the majority of stakeholders scored Completeness of information highly in most personas and DAMs, Completeness was revealed to have the lower end of the range (median 4.5, IQR 3.94-5) as opposed to Willingness to Use (median 5, IQR 4.67-5). Several stakeholders stated the need for more detailed information, specifically about the life of the persons with dementia, their history of employment, their hobbies, and other likes and dislikes. The hope was that knowing more about the life of that person could provide context for the robot designer to integrate a greater depth of knowledge into the conversations that the SAR could have with the patient, with the hope of engaging and distracting them, if necessary, and when agitation or risk of a fall might ensue. Furthermore, clinicians and nurse scientist found these personas and DAMs helpful in getting to know new patients more comprehensively. A nurse scientist and a lay caregiver both articulated this critical need to expand the details about the life of the person to

empathize more with the persons with dementia as greater details would provide information on what the patient loved to do.

One nurse scientist commented:

What can we do to get to know this person a little bit more, … what his life was like, … and talk to the people that are really important in his life, to build a further profile.

[Nurse scientist-1]

A nonnurse caregiver (facility nonnurse caregiver-1) found that speaking with the family about the activities the person with dementia engaged in with friends and community acquaintances was quite helpful. Another nurse scientist stated:

What did her life look like before dementia … She had a very famous career of design, and there is a part of her still designing … We make sure she has a choice of clothes, because those were the things that mattered to her, that’s sort of who she was …

[Nurse scientist-2]

Regarding training models which enable the SARs to interact with the patient, one dementia nurse reported the benefit of knowing what events brought joy to Norman, as that could spark a conversation and promote a behavior, like dancing, that may have been enjoyed in the past, or distract him from becoming agitated (practicing registered nurse-1). For a robot designer, the request was for understanding what George or Nolan used to do when they were alone (robot designer-1), as that would provide a greater understanding of how the robot can best interact with persons with dementia. Further, favorite TV shows or memorable representations of events may provide context to engage in questions of how he enjoyed them in his earlier life.

Finally, one family caregiver thought it was important to ask the family if their loved one knew why they were in the facility. For example:

what does the resident think that they’re there for?

[Family caregiver-1]

These social details would be very helpful to better understand the persons with dementia. However, aside from the social context of the lives of the persons with dementia, understanding their emotions and behavioral manifestations was needed to provide another dimension of understanding the life of the persons with dementia.

##### Understanding Present Emotions and Behaviors Helps Us Provide Better Care

In general, the stakeholders found the personas and DAMs very helpful in gaining an initial understanding of what emotions and behaviors were typically manifested at different times of day. For example, one psychiatrist remarked:

So I think it seems overall complete. But … there’s a little bit of information which can be added, more like how the persona Denise gets upset.

[Psychiatrist]

This was likewise expressed by a nonnurse caregiver and a nurse scientist about persons with dementia in general. There was also a need for more information on how personas express themselves emotionally. For Norman, specifically, there was interest in understanding if he was anxious or depressed, and if there was a diurnal variation in these emotions.

For Denise, there was a need for more details about why she was so apathetic, and what types of activities she engaged in, if any. As noted by another nurse scientist (nurse scientist-2), while not all persons with dementia are aggressive, underlying depression may mask the emotional reactions of persons with dementia and make them expressionless. Thus, a careful assessment of this type of confounding effect is needed (nurse scientist-2). Similarly, a dementia nurse expressed the need for more information about Nolan.

In his overview that, you know, the wife is heartbroken … he wants to go home. He thinks he’s not supposed to be here … So that, to me … is somewhat of an explanation for why he is rude … with the staff in addition to his diagnosis. But … what upsets him? ... That can give more of a holistic, kind of more psychosocial reason why the person behaves the way they do.

[Practicing registered nurse-2]

Finally, a dementia physician commented on the need to assess trauma as a potential cause of sleep problems, particularly among refugees, as a result of posttraumatic stress disorder (PTSD) or “night terrors” (geriatrician).

##### Understanding Sociocultural Practices Might Spark Interesting Conversation Between the Robot and the Person

One nurse scientist had comments on all 3 personas she reviewed about the need to get more detail about sociocultural issues, as it may help the caregiver jog the memory of the persons with dementia who are not able to communicate. She perceived that if the conversational AI systems to be developed included information about favorite activities, and religious and social practices of the person, a fruitful conversation might ensue between the SAR and the persons with dementia that could very well mitigate agitation. For example:

it could be helpful … to have, like, religion or spirituality noted, because oftentimes that could actually help someone become less agitated.

Finally, food constituted another critical sociocultural area of focus for this nurse scientist, which might bring joy and satisfaction to the person as something familiar from their earlier life.

### Comprehensive Assessment of Clinical History and Functional Capacities as Essential for the Provision of Safe and Effective Interaction for Persons With Dementia

This theme emphasizes the importance of detailed, multidimensional clinical assessments related to understanding the history of chronic disorders and medication history of the persons with dementia, as well as their mental and emotional health. Finally, being aware of the functional capabilities of the patient and specific caring approaches that are effective in calming them is key information that should be shared.

#### Understanding the History of Chronic Disorders and Medication Use to Care for Persons With Dementia

In general, the key stakeholders found that the personas and DAMs were quite helpful in making decisions about clinical care or for use in training the models to be integrated into future meaningful interactions between the robot and the patient. The sentiments expressed by both a nonnurse caregiver and a human-robot designer were “overall, it (personas and DAMs) has a good bit of information.” Nevertheless, more of the clinical-focused stakeholders, such as dementia nurses, nurse scientists, and a physician, were particularly interested in knowing more details about the types of chronic conditions the patients had been living with, including their medications. As expressed by a nurse scientist, getting a clear picture of what chronic diseases or disorders the patients have would be helpful in understanding symptoms they might be experiencing, and for some, could lead to agitation.

I wonder … if more information could be provided related to how he (Ralph) managed throughout his life, his chronic conditions, what stage is his heart failure? As his level of dementia is moderate to severe, … what has assisted him in going from an agitated state to a state of calm?

[Nurse scientist-1]

I think I also noticed that people, especially with the population that I’ve been working with, have oftentimes substance use histories, and … you can’t really tell if [their cognition] … is related to … active use, or issues related to cognitive impairment or decline.

[Nurse scientist-1]

In addition, a nurse was particularly interested in knowing more about the patient’s medication history, as that might explain why the patient was manifesting a certain way.

So, if a person was on Eliquis and had a bleed, I just wanted to know that’s why their skin has all these bruises …

[Nurse scientist-1]

#### Understanding Mental and Emotional Health to Better Offer Care

Stakeholders likewise focused on the need to have greater detail on the mental and emotional history of the patients, as that information was closely tied to their agitation. While a psychiatrist commented on the great detail already provided in the personas and DAMs, a curiosity for more detail was raised as being integral for how best to care for the patient.

It sounds like he’s anxious about his wife, and that’s nicely fleshed out here, … But is he anxious about other things too? … That will flesh out … who he is and how best to respond.

[Psychiatrist]

A nurse scientist was more curious about whether the patient had ever had a psychiatric disorder or a substance use disorder, as they may often give a clue to patient behavior. Similarly, a physician commented that knowing their background as a veteran could provide greater understanding of emotional reactions and risk for agitation and falls. For example:

But any older gentleman of certain ages could be from the Vietnam era which would trigger a PTSD question always as well, right?

[Psychiatrist]

Finally, making note of how the patient interacts day by day would be helpful to document in greater detail in an attempt to better understand the patient and keep them safe. For example:

Is he going for the door? Is he starting to throw things when the caregivers try and reassure him?

[Psychiatrist]

This provider argued that by understanding the interaction a little bit better, it could contribute to how one might approach the person with dementia and avert the risk of falls.

Another dementia nurse was also interested in understanding the reason why a patient gets upset.

… but again, … she cries, when a caregiver changes her or transfers her. Why is she crying? Is she getting triggered? Is there something here that I need to understand as to why she’s crying? So maybe we can … approach caring for in a different way… Maybe she only wants a female; maybe … there’s something in her background that we don’t know.

[Practicing registered nurse-2]

#### Functional Assessment

While one nurse considered the information provided as fine to make clinical decisions, she still needed more information about the type of support the persons with dementia might need for activities of daily living. These comments were likewise repeated by a nurse scientist. As the nurse caregiver questioned,

Is he able to walk without a wheelchair, or is he wheelchair-bound? Can he get up and walk?”

[Practicing registered nurse-1]

The need for assistive devices and the ability to walk are directly associated with fall risk, particularly as persons with dementia often forget to use their cane or wheelchair next to their beds when getting up.

This questioning about the risk of falls also translated to how the persons with dementia were getting to the facilities needed, such as the bathroom or the dining room where meals were served. To the facility caregivers, getting up in the middle of the night was often a significant risk, particularly when the patient was drowsy or disoriented and needed to use the bathroom. For example, as one dementia nurse questioned:

[What is] the toileting regimen scheduled? Does he have a commode? Does he have a urinal?

[Practicing registered nurse-2]

### Perceived Design Utility: Translating Person-Centered Knowledge Into Actionable Tools

A number of stakeholders perceived that the personas and DAMs provided great detail to make caregiver decisions (facility nonnurse caregiver-1 and robot designer-1). However, several stakeholders also provided specific suggestions for improving the design of the personas and DAMs in terms of clarity and usability.

#### Design Utility and Visual Design Considerations

Despite the fact that clarity was scored highly across all the personas and DAMs, several stakeholders also suggested some improved design features. Moreover, while a nurse scientist found the DAMs to be very helpful to get to know the persons with dementia before their first meeting, she felt adding an extra column to the DAM would be helpful to include what kind of calming language the caregiver found successful (nurse scientist-1). In addition:

“Using more bullet points would be helpful” to improve the visibility of the persona

[Nurse scientist-1]

For a dementia nurse (practicing registered nurse-2), modeling the persona to read similarly to a medical chart was thought to be easier for caregivers to use, while a nonnurse caregiver (facility nonnurse caregiver-2) thought the LLMs should include verbal suggestions to remind persons with dementia to use their walker, and to inform those attempting to get out of bed when it is unsafe, and help is on the way, encouraging them not to try to get up on their own.

A facility caregiver also pointed out a few areas that were incomplete or needed more detail. For example, in one DAM, under the category “lunch,” while it was reported that this person could not chew, the caregiver questioned what type of foods were being prepared for this person (family caregiver-1).

For a nurse with expertise in dementia (practicing registered nurse-2), a suggestion was made to incorporate health monitoring with an alert system if needed. For example, if the persons with dementia were on narcotics, tracking the respiratory rate would be important. For a nonnurse caregiver, where the robot would be placed in the room was brought up, as it would relate to the comfort of the persons with dementia (facility nonnurse caregiver-1).

#### Suggestions for Training the Robot on Verbal Conversations That Calm the Persons With Dementia

A facility nurse caregiver expressed the sentiment that the personas would be more useful and more caregivers would be willing to use them if the language a nurse used to calm a person with dementia was included and used in designing the conversational interface with the robot. She suggested:

Try soothing behaviors … like, if she hates being changed and she is incontinent, after you’re done, if you tell her you love her, which actually we do, it really calms her down … It’s just amazing, the things that make a difference … You just have to engage her and let her know that, you know, you love her.

[Nurse scientist-3]

For a psychiatrist, it was also important to recognize conversations that triggered agitation, such as trying to convince them that a heavy jacket might not be a good choice on a hot summer day. This provider surmised that it was more about decision-making and having the independence “to be able to know how to dress themselves.” Moreover, the psychiatrist offered a conversation that he felt would be very helpful in calming an agitated patient by means of distraction and engagement. [If Norman is trying to exit the room and says] “I want to go home, this isn't my home,” the first step is to kind of respond, to say, “You're safe here, it’s okay,” without arguing about whether it’s home or not, because that usually is a waste of time. To say, “You’re safe here, and your wife is around.” If the person then says, “No, I really want to go home, and is heading out the door,” one needs to make sure they’re safe and not to let them out, and then to redirect them, to move them towards something else. Say:

Hey, let’s go over here and look at what’s happening. This is interesting, isn’t it?

[Psychiatrist]

#### Nonconversational Approaches to Calm the Persons With Dementia

A number of stakeholders requested that nonconversational approaches that worked in calming the persons with dementia should be added to the personas and DAMs, so that the robot designers could incorporate these strategies in the SAR. These additional strategies would make the personas and DAMs even more useful and more widely used by caregivers. For example, TV was considered a possible intervention by one nurse scientist. However, she stressed that it was still important to understand which TV show the persons with dementia wanted to see, as putting on a TV show that was not wanted could also have negative consequences. For a nurse scientist, she contended that TV was not good for the brain or for depression, and it would be better if the robot designer focused on personalized activities that encouraged talking with others, playing a game, reminding the persons with dementia when family was arriving, etc. She emphasized that the robot should be used to engage and keep the persons with dementia grounded in time and space; thus, promoting a holistic benefit rather than just keeping them safe. For this nurse scientist, she described the sequence essential for both a caregiver and the LLMs to be trained on when a patient gets agitated. These include the following: (1) validating the patient’s feelings through active listening and repeating back what was said, (2) making sure they are safe, (3) redirecting the patient’s attention, and (4) doing something to relieve their anxiety, such as distraction (nurse scientist-2). Further, while touch was commonly stated as helpful, to a nonnurse caregiver, touch was to be carefully applied, as not all patients like to be touched.

For a psychiatrist, his suggestion was to add an area in the persona for what the SAR could do to engage residents more, such as using pictures, stories, etc (psychiatrist). He also added that “voice recordings and stories recorded by the family members will be calming for dementia patients.” Moreover, for an agitated person who constantly called for his wife, the psychiatrist added that, for Norman, the robot could also play a video of his wife saying, in a calm way, that he is safe and things are okay (psychiatrist). The psychiatrist also suggested using the employment history of the persons with dementia as a means of distracting them if they tend to get agitated. For example, if the resident was an engineer, give them a box of tools. He went on to add:

I’ve had many older veterans spending most of their afternoon taking carburetors … and deriving a lot of pleasure from it, and it’s distracting, and they have a good time with it. Okay, so, in his case, he was an aeronautics person, you know, a box of airplane parts would be great.

Similarly, a nurse scientist, clinical nurse, and psychiatrist each noted the importance of understanding that an attraction to pets may prove to be very helpful. Other examples included playing music that the resident loved, or native languages the resident may remember from childhood or younger adult years, which may likewise be calming:

could the robot play music that the person really loves, or if they speak another language, oftentimes, people who may have dementia actually remember their like native tongue. Could they speak … their language?

[Facility nurse caregiver-1]

## Discussion

### Principal Findings

This study used a mixed methods design to engage stakeholders involved in the care of persons with dementia or in the development of SARs, with the aim of assessing personas and DAMs using a modified PPS for potential application in clinical care or digital health solutions for persons with dementia. The descriptive, exploratory quantitative findings indicated that the personas and DAMs performed well in terms of credibility and clarity. Stakeholders from diverse fields, including clinicians and robot developers, expressed willingness to use these tools in their clinical practice or to inform the design of digital solutions for persons with dementia. However, the exploratory quantitative evaluation also suggested that the completeness and empathy constructs required further improvement to better reflect stakeholder needs and to more comprehensively represent the dementia population they work with.

Accordingly, the qualitative findings focused on identifying how the personas and DAMs could be refined to reflect stakeholders’ perspectives and provide a comprehensive representation of persons with dementia’s life trajectories and daily routines. Three major themes emerged from the qualitative analysis: (1) improvement of human-robot interaction training through greater knowledge of the personal context of persons with dementia, (2) comprehensive assessment of clinical history and functional capacities as essential for the provision of safe and effective interaction for persons with dementia, and (3) perceived design utility: translating person-centered knowledge into actionable tools.

To improve the empathy aspect of the personas and DAMs, stakeholders suggested that incorporating information on the sociocultural background and languages spoken by persons with dementia could enhance the personas and DAMs, enabling users of these tools to develop culturally informed solutions for this population. To support inclusive research practices, racial and ethnic diversity was intentionally sought among both the persona and DAM representations and the study participants. Participants were invited from various cultural backgrounds and were located in the United States (California and North Carolina) and Pakistan, with some US-based participants also having clinical experience in other countries. Stakeholders evaluated the tools through their own cultural lens and provided recommendations such as incorporating native languages, religious practices, and ethnic traditions that persons with dementia currently practice or have practiced throughout their lives. Embedding such cultural elements into personas and DAMs could support clinical settings in delivering culturally appropriate care, assist in matching caregivers to patients based on cultural compatibility [60], and guide digital health designers in developing solutions that foster greater empathy and relevance for this population [61-63]. This is supported by research consistently demonstrating that individuals tend to show stronger empathic responses toward those who share familiar cultural, linguistic, or ethnic characteristics, a phenomenon known as in-group empathy bias [64,65].

Willingness to Use received consistently high scores among both clinical and technical stakeholders. It serves as an integrative outcome measure, indicating whether the tools are perceived as sufficiently user-centered and empathy-inducing to be adopted in clinical and design practice. The Willingness to Use domain scores suggest that end-user willingness to engage with these tools may be favorable, warranting further investigation with larger and more diverse samples. The stakeholders also identified specific use cases for incorporating the personas and DAMs into their respective fields. Clinical stakeholders, in particular, viewed the personas and DAMs as valuable supplementary tools to complement existing medical records, providing health care providers and caregivers with contextual information that standardized clinical documentation often does not capture, including behavioral and emotional symptoms, agitation manifestations, life trajectory, and daily routines. This is especially relevant given that most persons with dementia present with multiple comorbidities, and an incomplete clinical picture may obscure accurate diagnosis and care planning. For instance, older adults with a military background may carry undiagnosed or undertreated PTSD, which can manifest as sleep disturbances or unanticipated behavioral outbursts during care delivery [66,67], symptoms that may be misattributed to dementia alone if the patient’s life history is unknown. Similarly, symptoms of depression, substance use withdrawal, or other treatable conditions may be overlooked or conflated with dementia-related cognitive decline, particularly in patients who can no longer communicate their subjective experiences to care providers. In such cases, the narrative richness of personas and DAMs, capturing a person’s background, habits, relationships, and emotional manifestations, may serve as a critical complement to clinical records, helping providers distinguish dementia-related symptoms from symptoms of undertreated comorbid conditions.

In addition, technical stakeholders, who reported high Willingness to Use scores, viewed the personas and DAMs as an opportunity, particularly for developers who may not have direct experience with this population, to immerse themselves in the lived experiences of persons with dementia, thereby fostering greater empathetic understanding. However, to be effective, these tools need to be truly representative of the diversity of persons with dementia and sufficiently detailed to provide the contextual data needed for personalizing conversational AI systems and designing appropriate human-robot interfaces for future SARs, ones that account for the cognitive impairment and possible physical disability of this population, thereby increasing the acceptability and feasibility of long-term SAR use and interaction [68].

To improve the personas and DAMs, stakeholders suggested incorporating the lived experiences of persons with dementia and their personal narratives of life trajectories and daily routines. This recommendation aligns with the current research trend toward involving persons with dementia in the co-design of digital health solutions [69,70] and supporting informed decision-making in their care [71,72]. However, given the cognitive impairments associated with dementia, conducting such participatory research presents notable challenges. These include difficulties in data collection, the likelihood that recall and narration may be affected by cognitive decline, and the risk of confabulation, whereby individuals unconsciously fill memory gaps with inaccurate or fabricated details [73]. Ethical considerations surrounding informed consent and participation capacity further complicate this approach. Nevertheless, this remains a valuable direction for future research, particularly in studies involving persons with mild dementia who retain sufficient cognitive capacity to meaningfully and reliably participate. Therefore, when collecting information about persons with dementia, it is important to first assess their awareness of their condition and the extent of their cognitive impairment. For persons with moderate to severe dementia, their social network, including family members, friends, and caregivers, represents the most appropriate and reliable source of information [74].

Persons with mild dementia may be able to contribute to the co-design and evaluation of persona-DAMs, and future studies should include them where appropriate, with careful attention to ethical considerations [75,76]. First, decision-making capacity should be assessed using validated tools [77,78], with regular reassessment throughout the study, and where capacity is insufficient, surrogate or legally authorized representatives should be involved in the informed consent process, while continuing to monitor the individual’s verbal and nonverbal expressions of assent or dissent. Second, privacy and data security require particular attention, as cognitive impairment may increase vulnerability to misuse of personal and financial information, fraud, and exploitation [79,80]. Accordingly, we recommend treating persona-DAMs as sensitive medical records subject to appropriate regulatory protections, and when used for research purposes, they should be fully deidentified and not traceable to individual patients, including avoidance of identifiable residential or care facility details. Third, SARs are conceived as tools to assist health care providers and caregivers in meeting the physical, emotional, and psychological needs of patients, particularly in settings of staff shortage and high caregiving burden, rather than as substitutes for human care [81]. However, potential emotional dependence and overreliance on SARs may reduce opportunities for human interaction and contribute to social isolation among persons with dementia [81]. Therefore, a balanced combination of SAR-mediated and human interaction should be considered when designing interventions. These ethical issues were beyond the scope of the present study but warrant further investigation involving persons with dementia, caregivers, clinicians, ethicists, and robotics engineers prior to broader implementation.

Grounded in the HCI conceptual framework [46], the findings suggest that personas and DAMs can help designers “foster empathy, better understand user needs, and develop new devices” [82], a goal that fundamentally aligns digital health solution design with the principles of PCC [83,84] in clinical settings. Both frameworks place the individual at the center of design and decision-making. Although cognitive impairment in persons with dementia often shifts formal decision-making authority to family caregivers and legal guardians [73,85], embedding individual preferences into personas and DAMs honors the person’s identity and values, enabling clinicians and caregivers to deliver personalized care and developers to design digital health solutions that reflect the needs of the whole person. Reflecting this alignment, several stakeholders recommended enhancing the Completeness domain of the personas and DAMs by incorporating both conversational and nonconversational calming and coping strategies, such as topics of interest for validation-based conversation, music therapy, and interventions informed by the person’s occupational history, that could guide nonpharmacological interventions for mitigating states such as agitation. In this way, the personas and DAMs serve as a tool that translates a holistic understanding of the individual into actionable guidance for both clinical care and the development of socially assistive technologies.

### Limitations

We acknowledge several limitations of this study. First, the small sample size (N=15) and small subgroup sample sizes may limit the ability to conclude representative of each professional stakeholder category. Also, limitations in information power within stakeholder professional subgroups are acknowledged. Future studies should recruit larger numbers of participants per professional category to improve representativeness. However, it should be noted that the full qualitative sample provided adequate information power to address the overall study aim.

Second, the sampling method was purposive, and participants were drawn from the research team’s professional network, introducing an element of convenience sampling that may limit the generalizability of the findings to broader populations of dementia care stakeholders. Participants who accepted the invitation may have had a preexisting interest in or familiarity with the research topic, potentially resulting in more favorable evaluations than would be observed in a broader, randomly selected sample. Additionally, the nonresponse of the 4 individuals who declined the invitation may reflect limited interest in or skepticism about the applicability of the tools, which could represent a potential source of selection bias. Further, as participants selected persona-DAM pairs based on familiarity and perceived relatability rather than through random assignment, any observed differences could partly reflect the characteristics of the participants who evaluated particular persona-DAM pairs rather than differences attributable to the personas themselves. Accordingly, quantitative scores were pooled across persona-DAM evaluations and were not reported or compared at the level of individual personas and DAMs. This nonrandom selection process limits comparisons and generalizability across individual personas and DAMs.

Third, as participants each rated multiple personas and DAMs (an average of 2.4, SD 0.63, persona-DAM pairs per participant), the data contain nonindependent observations. Therefore, we report participant-level descriptive statistics to assess whether the findings were influenced by participants contributing multiple evaluations. However, the quantitative component is reported for exploratory and descriptive purposes only.

Fourth, persons with dementia were not included as study participants. The current study also did not undertake a comprehensive ethical evaluation of SAR deployment in this population. Key ethical considerations related to involving persons with dementia as research participants or co-designers include cognitive impairment and decision-making capacity, reliance on surrogate decision-making for informed consent, and protection of privacy and data security. These issues warrant dedicated investigation before broader implementation of these technologies in dementia care. In light of these considerations, the limited inclusion of perspectives from persons with dementia in research and the co-design of digital health interventions represents an important gap that should be addressed in future studies.

Finally, given the small sample size, formal psychometric validation of the modified PPS, including reliability and validity testing for this specific population and the adapted scale items, was not conducted in the current study. Psychometric evaluation of the modified PPS for use with dementia care stakeholders remains an important direction for future research.

### Conclusion

This mixed methods study provides early-stage evidence that personas and DAMs are perceived as clinically and technically relevant tools for supporting the care of persons with dementia. Clinical stakeholders recognized their potential to complement existing clinical documentation by providing a holistic view of the personal, medical, behavioral and emotional, sociocultural, and life trajectory aspects of the person with dementia. Technical stakeholders recognized their value in designing personalized digital health solutions, including SARs, by better understanding this patient population and fostering empathy in developers with minimal exposure to this population. Across stakeholder groups, willingness to use these tools in their respective fields was expressed.

Ultimately, this study lays the groundwork for integrating human-centered design tools into dementia care and technology development, to personalize intervention recommendations based on patient preferences and interests [86], specializing conversational AI for empathetic interaction aligned with the patient’s cognitive status [87], and increasing the acceptability and long-term feasibility of digital health and assistive technologies for persons living with dementia.

## Supplementary material

10.2196/85651 Multimedia Appendix 1 Personas and daily activity maps (DAMs).

10.2196/85651 Multimedia Appendix 2 Modified Persona Perception Scale (PPS).

10.2196/85651 Multimedia Appendix 3 Interview Guide.

10.2196/85651 Checklist 1 Consolidated Criteria for Reporting Qualitative Research (COREQ) Checklist.

## Abbreviations

COREQ: Consolidated Criteria for Reporting Qualitative Research
DAM: daily activity map
e-gift: electronic gift
HCI: human-computer interaction
IRB: Institutional Review Board
LLM: large language model
MARIO: Managing Active and Healthy Aging with Use of Caring Service Obots
PARO: Personal Assistive Robot
PCC: person-centered care
PPS: Persona Perception Scale
PTSD: posttraumatic stress disorder
SAR: Socially Assistive Robot
UCD: user-centered design
UI: user interface
UX: user experience
