# Supplementary material for: Evaluation of Personas and Daily Activity Maps to Inform Clinical Care and Socially Assistive Robot Training and Design for Persons With Dementia: Mixed Methods Study
Source: J Med Internet Res. 2026 Oct 28;28:e85651. doi: 10.2196/85651 (PMC13718279; doi:10.2196/85651)
Supplement: Supplementary file 1 [file jmir-v28-e85651-s001.pdf]

Multimedia Appendix 1. Personas and Daily Activity Maps for Nine Pseudo Patients Living with Dementia.

Denise

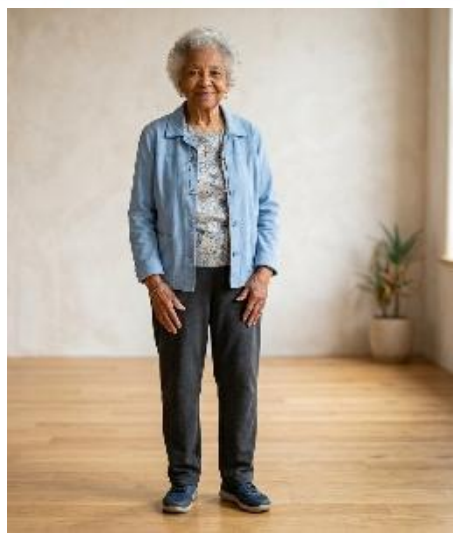

Overview
87 years old – moderate dementia.
Keeps to herself
Somewhat confused – "where am I?"
Military background (husband, son, ...)

Level of Dementia
Moderate dementia.

Health Condition
Generally good

Chronic Diseases
High blood pressure

Hearing
No hearing issues

Eyesight
Good.

Mobility
Walks and goes to bathroom hersel

Conversational Ability
Can carry on a conversation.
No hearing issues.

Entertainment & Communication Devices Used
TV
Landline phone

Behaviors to Watch For
Confusion

Personality
Sweet personality
Calm, not aggressive, modest
Needs redirection occasionally

Interests
Loves watching American Classic TV Network (e.g. Andy Griffith, MASH)
Looks at old photos with daughter
Loves sweets

Sleep Quality
Sleeps well on recliner couch, in upright position.
Needs very warm room, electric blanket

Cultural Issues
African-American
Prefers female caregiver

Medications
Anti hypertension
Cholesterol
Vitamins
Constipation

Emotions
Calm
Non-aggressive

What Makes Her Happy
Watching American Classic TV Network (e.g. Andy Griffith, MASH)
Looking at old photos with daughter

What Upsets Her
Being cold

Caregiver Interventions
Manage medications and meals
Help her to the shower

Personal Stories
Military background (husband, son)

Denise

Activities	Sleep 7 PM	Wake-Up	Breakfast	Morning Activities	Lunch	After Lunch Activities	Afternoon Nap	Dinner 5-6 PM	Turndown Time 6-7 PM
Action(s)	Sleeps well	• Given bed bath by staff.<br>• Bed linens changed.<br>• Walks into dining room herself	• Eats whatever is given.<br>• Given meds.<br>• Walks to the TV room.	• Stays in her room bundled up<br>• Sometimes watches American Classic TV Network (e.g. Andy Griffith, MASH) in community room	• Eats (lots of sweets for dessert).<br>• Given meds.<br>• Walks to the TV room.	• Watches American Classic TV Network (e.g. Andy Griffith, MASH).<br>• Looks at old photos with daughter.<br>• Stays in her room bundled up.	• Sleeps either in her recliner or in bed.<br>• Is bundled up because she is always cold.	• Eats.<br>• Given meds.	Gets ready for bed.
Health Condition	1.Moderate dementia<br>2.Hypertension	Good	Good	Good	Good	Good			
Emotional State	Calm, not aggressive	Mostly flat affect	Mostly flat affect	Mostly flat affect	Mostly flat affect	Happiest when daughter visits.		Mostly flat affect	
Caregiver Intervention		• Caregiver wakes and prepares her for the morning.<br>• Caregiver takes blood pressure and heart rate before giving meds.<br>• Caregiver places call button within reach at all times.			• She walks to the dinner table herself.<br>• Feeds herself lunch.			• She walks to the dinner table.<br>• Feeds herself dinner.	• Caregiver cleans and prepares her for bed.
Device Touchpoint	Call button available at all times and aware of using.	Call button available	Call button available	Controls TV with remote	Call button available	Call button available	Call button available		Motion detector in room
Opportunities for Robot-Human Interaction		Heart rate and BP				• Show old photos<br>• Daughter videos and still photos.<br>• Reminisce about military (husband and son were in military).			Robot can detect her transitions, actions, and facial expressions and react to triggers as appropriate.

Jolene

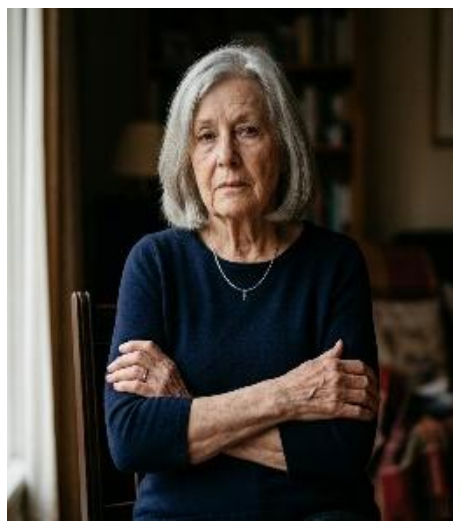

Overview
Caucasian woman about 82 years of age
She is depressed and generally expressionless.

Level of Dementia
Mild

Health Condition
Multiple Sclerosis

Chronic Diseases
Hypertension
Multiple Sclerosis

Hearing
OK

Mobility
Can use her lower extremities and move herself to the W/C with standby assist.

Conversational Ability
Expressionless and monotone conversation.

Entertainment & Communication Devices Used
TV
Motion detector

Behaviors to Watch For
Understands what you're talking about. No adverse behaviors.
If she frowns – possible indication she does not like what is happening – but still does not state what she prefers.

Personality
Before hospitalization, very outgoing, enjoyed dancing. Often expressionless. She understands conversations but provides monotone response. When asked a question, her typical answers are "fine" "ok" or "yes".
Former elementary teacher.

Interests
Loves watching American Classic TV Network (e.g. Andy Griffith, MASH)

Sleep Quality
OK

Cultural Issues
No cultural preferences articulated

Medications
Lexapro

What Makes Her Happy
She has no preferences; no expression when son visits.

What Upsets Her
Nothing gets her upset

Caregiver Interventions
"An easy patient to care for"

Jolene

Activities	Sleep 7 PM	Wake-Up	Breakfast	Morning Activities	Lunch	After Lunch Activities	Afternoon Nap	Dinner 5-6 PM	Turndown Time 6-7 PM
Action(s)		• Gets cleaned<br>• Wheeled to the dining room.	• Eats.<br>• Given meds.<br>• Wheels to the TV room.	• Watches TV with other residents in the living room for 45-60 minutes.<br>• Wheeled back to her room and watches TV there	• Wheeled to the lunch room.	• Wheeled into living room where she eats sweets<br>• Watches TV in living room<br>• Husband or son visits and they converse	• Wheeled into bed<br>• Takes nap	• Wheeled to the dining room.	
Health Condition									
Emotional State	Sleeps well	Expressionless	Calm Expressionless	Expressionless		Expressionless		Expressionless	
Caregiver Intervention		Caregiver wakes, cleans, dresses, and performs ADL activities with her.	Wheels her into dining room.	Caregiver wheels her to a chair/couch.	Wheels her into dining room.		Wheels her into bedroom.		Caregiver cleans and prepares her for bed.
Touchpoint: Device									
Opportunities for Robot-Human Interaction				• Robot recalls her time as an elementary teacher.<br>• Robot detects emotions.					

Nolan

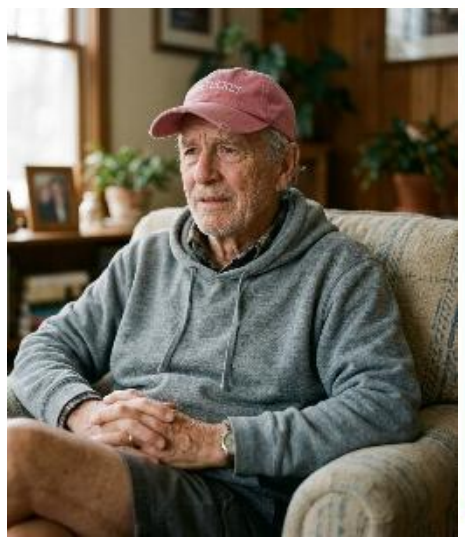

Overview
76 year old Caucasian male with severe dementia. Worked as an engineer.
Has a wife, she is heartbroken about his condition and inability to take care of him. Never aggressive prior to being diagnosed with dementia.
One grown son. No pets
3rd fall at home; leading to the need to bring him to the facility.
Asserts he is not supposed to be in the residence. Wants to go home.

Health Condition
Overall generally healthy
Sleep quality is typically poor

Chronic Diseases
Progressive dementia - Mostly cognitive. Eyesight - needs glasses

Hearing
Fine

Mobility
unstable with walker: fall risk

Conversational Ability
Talks periodically with other residents or caregivers
70% of the time, words do not make sense.

Entertainment & Communication Devices Used
Motion detector in room
Bed alarm, W/C alarm.
Gets up with physical therapy so muscles do not atrophy. But very risky as he is a fall risk.
Put into W/C once a day as requested by wife

Behaviors to Watch For
Believes he is only there temporarily. Wife cannot take care of him at home. Facility is thus unfamiliar and it is triggering.
Tells staff to shut their mouth, leave him alone when bathing him.

Personality
Intermittently fluctuates between calmness and agitation coinciding with confusion.
When experiencing these behavioral problems, gets aggressive with caregiver.
Agitation changes quickly.

Interests
Watches TV with other residents
Asks for his wife
Likes Fox news and Andy Griffith show

Sleep Quality
Sundowning; being treated with meds

Cultural Issues
No cultural preferences articulated

Medications
Atavan for agitation, Haldol and Seroquel
Seroquel doubled at night due to sundowning

What Makes Him Happy
Happy when wife is at the facility

What Upsets Him
He will only take meds when wife is at facility. Otherwise, he does not take them. When he gets upset, his wife gets emotional.

Caregiver Interventions
Staff use calm language to sooth him.
Staff hold his hand so he does not swing at them. Tell him, "you're ok; we are here to help you".
Being evaluated by psych nurse to get his meds adjusted as requested by wife.

Nolan

Activities	Sleep 7 PM	Wake-Up	Breakfast	Morning Activities	Lunch	After Lunch Activities	Afternoon Nap	Dinner 5-6 PM	Turndown Time 6-7 PM
Action(s)	Sleep quality is typically poor	• Caregiver wakes, cleans, dresses, and performs ADL activities with him<br>• Wheeled to the dining room.<br>• Wears a Posey vest	• Eats. Needs to be spoon-fed on some days.<br>• Given meds.<br>• Wheeled to the TV room.	• Watches TV.	• Eats. Needs to be spoon-fed on some days.<br>• May talk to Doug<br>• Given meds.	• Wheeled to the TV room.<br>• Watches TV<br>• Wears a Posey vest	• Wheeled to the bed.<br>• Takes a nap.	• Eats. Needs to be spoon-fed on some days.<br>• May talk to Doug<br>• Given meds.	• Watches TV in the Living Room.<br>• Wheeled to his bed.
Health Condition	• Severe dementia<br>• Overall generally healthy<br>• Sleep quality is typically poor			Sedated		Sedated	Sedated		Sedated
Emotional State		• Intermittently fluctuates between calmness and agitation coinciding with confusion.<br>• Agitation changes quickly.		• Cries.<br>• "I don't want to be here".		• Cries.<br>• "I don't want to be here".			
Caregiver Intervention		• Has behavioral problems, gets aggressive with caregiver (punches caregiver).<br>• Use calm language to sooth him.<br>• Staff hold his hand so he does not swing at them.			• Caregiver transfers to wheelchair and wheels him to dining table.			• Caregiver transfers to wheelchair and dining table.	• Caregiver transfers him to a wheelchair and his bed.
Device Touchpoint		• Wheelchair has a pad alarm.		• Wheelchair has a pad alarm.	• Wheelchair has a pad alarm.	• Wheelchair has a pad alarm.			• Wheelchair has a pad alarm.
Opportunities for Robot-Human Interaction		Deescalate agitation	Deescalate agitation	Deescalate agitation	Deescalate agitation	Deescalate agitation		Deescalate agitation	

Ralph

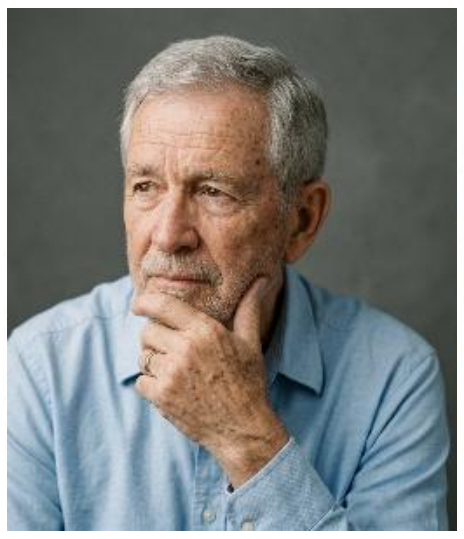

Overview
78 years old. Fell at home and broke right hip, went to rehab, now at B&C. Has prostate cancer and heart disease. Very calm, grew up in Hawaii, did surfing. Wife passed a year ago. He gets up on his own and poses a significant fall risk.

Level of Dementia
Significant dementia.

Health Condition
Cognitive decline.
Incontinent (both urine and stool)
Chronic Diseases
Prostate cancer
Heart disease
Gout (chronic pain)
Hearing
Wears hearing aids
Eyesight
Good. Wears glasses
Mobility
Uses walker. He will get up without caregiver assistance-potential for falls.

Conversational Ability
Appears to be able to carry on conversations, but often he really doesn't understand what is being said.

Entertainment & Communication Devices Used
TV
Mobile phone

Behaviors to Watch For
Impulsive. He gets up on his own and moves around without his walker - fall risk. Hurts his hands while trying to get out of bed. Aggressive towards caregivers when they are trying to dress him-grabs their hands and slaps. Gropes caregivers.

Personality
Severe confusion. Impulsive. Often calm but confused. After he broke his hip and was hospitalized he began to exhibit more cognitive decline and fell into delirium. Likes to be around people - carefree.

Interests
Outdoors (sit in sun)
Likes to be among people
Likes sweets

Sleep Quality
Poor sleep quality. Severe sundowning.

Cultural Issues
Jewish faith (not practicing)

Food Restrictions
No pork products

Medications
Seroquel
ABHR Cream
Ativan
Haldol
Tramadol
Trazodone
Norco

Emotions
Anger
Surprise

What Makes Him Happy
Sweets (ice cream, desserts)
Companionship

What Upsets Him
Being isolated.
Restricting him.
He misses being at work.

Caregiver Interventions
Caregivers cannot monitor James all the time - they have other residents to watch. Caregivers need to be patient and calm him down.

Personal Stories
Used to work at nuclear power plant.
Surfing days in Hawaii.
Family and kids.
Concerned about his daughter who just went through divorce.

Ralph

Activities	Sleep 7 PM	Wake-Up	Breakfast	Morning Activities	Lunch	After Lunch Activities	Afternoon Nap	Dinner 5-6 PM	Turndown Time 6-7 PM
Action(s)	• Goes to bed late – 10–11 PM.<br>• Sleep quality is typically poor – severe sundowning.	• Caregiver wakes, cleans, dresses, and performs ADL activities with him.<br>• Wheeled to the dining room.	• Eats. Food is pureed because he has no swallowing reflexes.<br>• Spoon fed.<br>• Given meds.<br>• Wheeled to the TV room.	• Watches TV.<br>• Goes back to his own room with door open so that caregivers can observe him.	• Eats. Food is pureed because he has no swallowing reflexes.<br>• Spoon fed.<br>• Given meds.	• Wheeled to the TV room.<br>• Watches TV.	• Wheeled to the bed.<br>• Takes a nap on some days and on other days can't fall asleep.	• Eats. Food is pureed because he has no swallowing reflexes.<br>• Spoon fed.<br>• Given meds.	• Watches TV in the Living Room.<br>• Wheeled to his bed.
Health Condition	• Severe dementia<br>• Cognitive decline.<br>• Incontinent (both urine and stool)<br>• Chronic Diseases<br>• Prostate cancer<br>• Heart disease<br>• Gout (chronic pain)								
Emotional State	• Intermittently fluctuates between calmness and agitation coinciding with confusion.<br>• Agitation changes quickly. NOTE – this persists all day.			Sedated		Sedated			Sedated
Caregiver Intervention		• Has behavioral problems, gets aggressive with caregiver (punches caregiver, took off clothes).<br>• May try to stand on his own – caregiver needs to monitor and intervene.		• He may lash out at caregiver if the caregiver does something he does not like.	• Caregiver transfers to wheelchair and wheels him to dining table.	• Keep him in the living room longer than other residents in order to monitor him.		• Caregiver transfers to wheelchair and dining table.	• Caregiver transfers him to a wheelchair and his bed.
Device Touchpoint									
Opportunities for Robot-Human Interaction		• Deescalate agitation<br>• Play videos or voice of son or daughter.	• Deescalate agitation<br>• Play videos or voice of son or daughter.	• Detect when he gets up on his own (fall risk).	• Deescalate agitation<br>• Play videos or voice of son or daughter.	• Detect when he gets up on his own.<br>• Deescalate agitation		• Detect when he gets up on his own.<br>• Deescalate agitation	

George

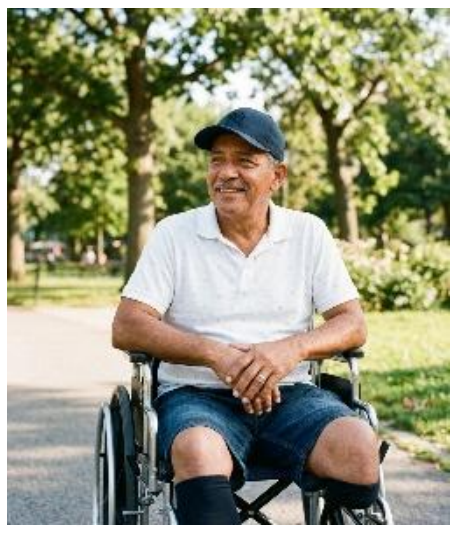

Overview
62 years old. Frequently lashes out at caregivers when wife is not there: well-behaved when she is there. Manipulative personality. Is under the impression that B&C staff is incompetent and expects staff to do whatever he commands - feels entitled.

Level of Dementia
Early stage dementia.

Personality
Angry at the world. At first he is very cordial but then his behavior deteriorates into frustration. He thinks he knows how to take care of himself better than caregivers and insists that he knows how much medication to take at meals.

Interests
Watches TV
Video games
iPad

Sleep Quality
Poor sleep quality, mostly attributed to opioids he takes. Will sleep for short periods (up to an hour), wakes up frequently.

Health Condition
Generally poor
Due to diabetes, he lacks digestive enzyme so must eat 8x day, resulting in frequent stooling/poor hygiene.
Chronic Muscle spasms

Chronic Diseases
Chronic Diabetes Type I
Congestive heart failure
Hypertension.
Neuropathy (2 legs amputated below knee)

Hearing
Good

Eyesight
Good. Wears glasses

Mobility
In wheelchair-transfer dependent
Bilateral prosthesis

Medications
Insulin
Creon (helps digest food)
Morphine
Gabapentin
Tramadol
Norco
Lasix

Conversational Ability
Very alert when awake.
Does not seek companionship or conversation with other residents.

Entertainment & Communication Devices Used
TV
Mobile phone
iPad (play video games and Face Times daughter on iPad)

What Makes Him Happy
Family visits from wife and son.
Watching TV.

What Upsets Him
B&C staff: He feels they are incompetent and expects staff to do whatever he commands - feels entitled.

Behaviors to Watch For
Gets agitated when he doesn't get immediate response from caregiver.
When wife is not present will lash out at caregivers. Uses foul language.
Caregivers must give insulin - need to ensure that correct amounts are given. He will insist he knows the appropriate dose.

Caregiver Interventions
Caregivers must give correct medication whenever he eats: correct amount of insulin and Creon every time he eats (which is about 8x daily). Then must assist with incontinence: sometimes he can't control himself.
Caregiveres cautious about dispensing too many opiods: He often says he is in pain.

Personal Stories
None.

George

Activities	Sleep 7 PM	Wake-Up	Breakfast	Morning Activities	Lunch	After Lunch Activities	Afternoon Nap	Dinner 5-6 PM	Turndown Time 6-7 PM
Action(s)	Goes to bed.	• Tells his caregiver what he plans to eat (how much carbs needed) and the caregiver determines additional insulin needed.<br>• Wheels to the dining room.	• Eats.<br>• Given meds.<br>• Wheels to the TV room.	• Watches TV.<br>• Plays video games on an iPad.	• Tells his caregiver what he plans to eat (how much carbs needed) and the caregiver determines additional insulin needed.<br>• Wheels to the dining room.	• Pet therapy.<br>• Uses iPad.<br>• Takes shower in shower chair.	• Wheels to the bed.<br>• Takes a 2-hour nap.	• Tells his caregiver what he plans to eat (how much carbs needed) and the caregiver determines additional insulin needed.<br>• Wheels to the dining room.<br>• About 45 minutes after meds becomes restless and complains of pain.	• Watches TV in the Living Room.<br>• Wheels to his bed.
Health Condition	• Snacks and his insulin level must be recalculated.<br>• Complains of pain and seeks opioids.<br>• Lays in bed (restless) and experiences difficulty going to sleep.	Double amputee	Double amputee	• About 45 minutes after meds becomes restless and in pain (back).<br>• Diabetes clouds his mind; he asks for something but changes his mind.<br>• He is incontinent and requires diaper change.	• Diabetes<br>• Double amputee	Double amputee		• Back pain<br>• Diabetes clouds his mind; he asks for something but changes his mind.<br>• He is incontinent and requires diaper change.<br>• Double amputee	Double amputee
Emotional State		😡 ☹️	😡 ☹️	😡 ☹️	😡 ☹️	😡 ☹️		😡 ☹️	😡 ☹️
Caregiver Intervention		• Caregiver checks blood sugar to determine level of insulin needed.<br>• Caregiver transfers to wheelchair and dining table.		Caregiver changes diaper.	• Caregiver checks blood sugar to determine level of insulin needed.<br>• Caregiver transfers to wheelchair and dining table.	Caregiver transfers him to the shower.		• Caregiver checks blood sugar to determine level of insulin needed.<br>• Caregiver transfers to wheelchair and dining table.<br>• Caregiver changes diaper.	• Caregiver checks blood sugar to determine level of insulin needed.<br>• Caregiver transfers him to a wheelchair and his bed.
Device Touchpoint			He monitors his glucose levels using Dexcom.						
Opportunities for Robot-Human Interaction				Robot plays games with him.<br>Robot displays pictures of son.		Robot prevents triggering.			

Billy

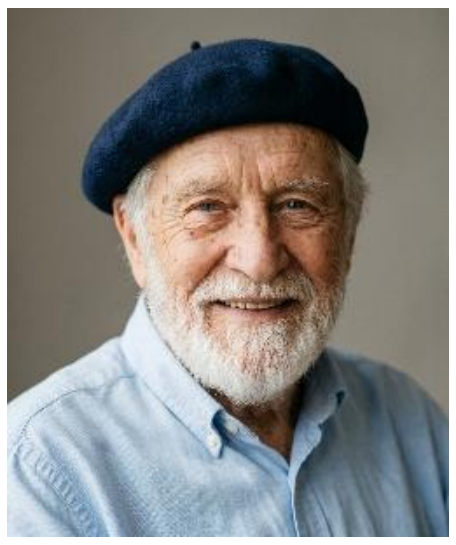

Overview
82 years old. Previously owned his own business (motorcycle and auto repair). He is very empathetic.

Level of Dementia
Mild to moderate

Health Condition
Generally good. Fell at home several times before coming to B&C.
Chronic Diseases
Hypertension.
Hyperlipidemia.
Hearing
Good
Eyesight
Good. Wears reading glasses
Mobility
Can walk with assistance (walker).
Can go to bathroom himself.

Conversational Ability
He likes to talk.
Likes to interact with other residents.

Entertainment & Communication Devices Used
TV
Mobile phone
iPad (play video games and Face Times daughter on iPad)

Behaviors to Watch For
Make sure he doesn't fall - doesn't always use his walker (mix of forgetting and wish to stay independent)

Personality
Very outgoing. Calm, generally happy in good spirits. He is very empathetic. Wants to go home but realizes that he is better off in B&C.

Interests
Likes to leave B&C to visit with family.
Video games.
Ice cream and sweets.

Sleep Quality
Sleeps throughout the night (6 hours REM). No meds requried.
Takes short power naps during day.

Cultural Issues
Prefers Spanish language
Food Restrictions
None

Medications
Zantac
Senna

What Makes Him Happy
Conversations with other residents
Speaking Spanish w/caregivers.
Singing.
Eating sugery and Mexican foods.

What Upsets Him
Nothing really upsets him

Caregiver Interventions
He does not require much caregiver intervention
Converse in Spanish
Intervene for fall risk

Personal Stories

Billy

Activities	Sleep 7 PM	Wake-Up	Breakfast	Morning Activities	Lunch	After Lunch Activities	Afternoon Nap	Dinner 5-6 PM	Turndown Time 6-7 PM
Action(s)	Goes to bed.	• Cleans himself.<br>• Only needs assistance with showering (prefers male caregiver).<br>• Walks to the dining table.	• Eats. (likes Mexican food)<br>• Given meds.<br>• Walks to the TV room.	• Watches TV.<br>• Plays video games on iPad.<br>• Calls family.<br>• Loves to visit and talk with other residents.	• Eat (lots of sweets for dessert).<br>• Be given meds.<br>• Walks to the TV room.	• Watches TV.<br>• Interacts with an iPad.<br>• Participates in the Music therapy/Pet therapy (alternative).<br>• Showers.	Takes a short power nap. (~ 15 minutes)	• Eats.<br>• Given meds.<br>• Watches a Special TV show.	Gets ready for bed.
Health Condition		• Hypertension<br>• High cholesterol<br>• Mild dementia							
Emotional State	😁	😑	😁	😑<br>Homesick	😁	😁	😁	😑	😑
Caregiver Intervention		• Blood pressure check.<br>• Only needs assistance with showering (prefers male caregiver).	Caregiver prepares food.						
Device Touchpoint		Heart rate, blood pressure, and pulse oximetry check.		• Watches TV.<br>• Plays video games on iPad.<br>• Calls family.		• Watches TV.<br>• Plays video games on iPad.<br>• Calls family.		Watches TV.	
Opportunities for Robot-Human Interaction		Assess fall risk and intervene if necessary.	• Reminds him to use his walker.<br>• Assess fall risk and intervene if necessary.	Assess fall risk and intervene if necessary.	Assess fall risk and intervene if necessary.	Assess fall risk and intervene if necessary.	Assess fall risk and intervene if necessary.	Assess fall risk and intervene if necessary.	Assess fall risk and intervene if necessary.

Norman

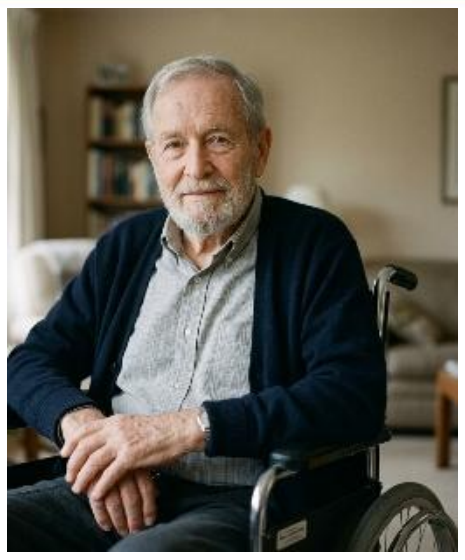

Overview
84 years old. Worked for Boeing for over 40 years. Engineer then manager - responsible for contracts. He and wife own a business (aircraft parts).

Level of Dementia
Moderate to Severe

Health Condition
Hypertension. Has pacemaker due to heart failure. Later diagnosed with dementia.
Chronic Diseases
Hypertension
Heart failure
Hearing
Good
Eyesight
Wears glasses
Mobility
Wheelchair

Conversational Ability
He likes to talk.
Articulate but repetitive stories.
Some days not interactive - single syllables - fragmented language.

Entertainment & Communication Devices Used
TV

Behaviors to Watch For
He wants to take care of wife - need to reassure him that she is OK. He tries to walk out front door to leave to take care of wife.
He starts to ask for his wife repeatedly - when tone and pitch start to change then agitation triggered.
When walking - pacing back and forth.

Personality
When first came to facility was calm. Soon got aggressive. Wanted to call wife repeatedly. Wife couldn't handle him at home. Falls at home (3x) before seeking help.

Interests
Collects Disney characters.
Loves dogs and cats

Sleep Quality
Sleeps throughout the night.
Will sleep through day - caregivers need to get him up to eat.
Before medication - had severe sundowning syndrome.

Medications
Risperdal
Serouquel
Haldol

What Makes Him Happy
Animals - pet therapy
Visits from wife
Eating sugery food-e.g. Cheerios
Watching animal TV shows (Youtube stream repeating)

What Upsets Him
Gets upset when caregivers don't have time to hear his stories.
Gets upset when he can't talk to wife. She only wants 1 call per week.

Caregiver Interventions
Calming language
Touching
Telling him that Dusty and Rusty are coming to visit him.
Call wife (Joyce) to speak with him
Let him know cats D&Y are safe

Personal Stories
Remembers grandfather's pet store
Favorite cats: Dusty and Rusty
Working with military during Boeing career (C-17, ICBM)

Norman

Activities	Sleep<br>7 PM	Wake-Up	Breakfast	Morning Activities	Lunch	After Lunch Activities	Afternoon Nap	Dinner<br>5-6 PM	Turndown Time<br>6-7 PM
Action(s)	Goes to bed.	• Given bed bath by staff.<br>• Bed linens changed.<br>• Wheeled to the dining room.	• Eats whatever is<br>• given.<br>• Given meds.<br>• Assisted to go to the TV room.	• Watches animal TV channels.	• Eats (lots of sweets for dessert).<br>• Given meds.<br>• Assisted to go to the TV room.	• Watches TV.<br>• Colors painting books.<br>• Does puzzles and Bingo.<br>• Receives Physical therapy (give pain meds 30 min prior).<br>• Participates in Music therapy/Pet therapy (alternating).<br>• Bed bath.<br>• Visited by a healthcare provider (RN).	Takes a nap. (1-2 hrs.)	• Eats.<br>• Given meds.	Gets ready for bed.
Health Condition	• Moderate dementia<br>• Hypertension<br>• Sundowning	• Generalized weakness	Generalized weakness	• Poor vision, requires glasses.<br>• Hearing impairment.	Generalized weakness	Generalized weakness			Sundown Syndrome
Emotional State				Happiest when wife visits.<br>When his pets come to visit.		Happiest when wife visits. When his pets come to visit.			
Caregiver Intervention	• Caregiver checks for incontinence several times a day and cleans as needed.	• Caregiver wakes and prepares him for the morning.<br>• Caregiver takes blood pressure and heart rate before giving meds.<br>• Caregiver places call button within reach at all times.	• Caregiver wheels him to the dinner table.<br>• Caregiver spoon feeds him.	Caregiver wheels him to a chair/couch.	• Caregiver wheels him to the dinner table.<br>• Caregiver spoon feeds him.	Caregiver gives him a bed bath again if needed.	Caregiver cleans his incontinence.	• Caregiver wheels him to the dinner table.<br>• Caregiver spoon feeds him.<br>• Caregiver assesses whether he has Sundowning Syndrome and begins getting agitated.	• Caregiver cleans and prepares him for bed.<br>• Caregiver gives him liquid melatonin to help sleep better.
Device Touchpoint	Call button available at all times and aware of using.	Call button available	Call button available	• Controls TV with remote<br>• Volume of TV is high due to hearing loss.<br>• Call button available	Call button available	Call button available	Call button available		Motion detector in room
Opportunities for Robot-Human Interaction		Heart rate and BP	• Loves to share personal stories<br>• Robot shows videos of Rusty and Dusty to calm him.	Loves to share personal stories.<br>Likes to talk to Joyce on the phone.	Loves to share personal stories.	Loves to share personal stories.			Robot can detect his transitions, actions, and facial expressions.

Rachel

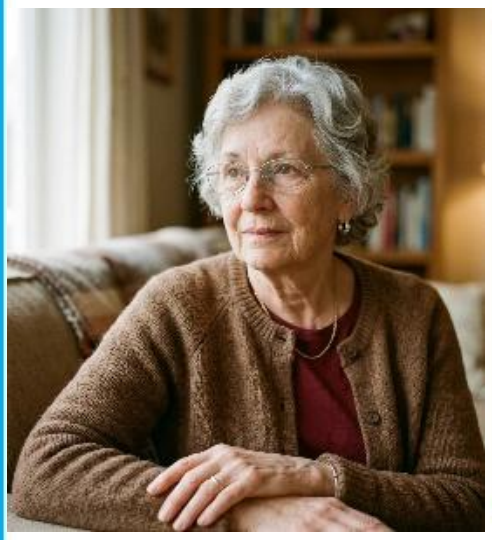

Overview
80-year old woman with severe dementia. She requires a wheelchair and cannot walk on her own.

Level of Dementia
Severe

Health Condition
Overall generally healthy
Sleep quality is typically poor
Blood pressure:
...

Chronic Diseases

Hearing
Hard of hearing

Mobility
Wheelchair

Conversational Ability
Generally difficult to have a conversation with her. However, her conversational ability improves when her son is visiting.

Her way of communicating is through cries, screams, and making noises.

Entertainment & Communication Devices Used
TV

Behaviors to Watch For
When she wakes up, she often doesn't know where she is. She will call out to the staff and if no one responds quickly she will rattle her bedrails.

Personality
She is often sad.
She can become extremely distraught when disoriented.

Interests
She looks forward to visits from her son.

Sleep Quality
Early riser (5 am)
Sundowning ?

Medications
Risperdal
Tramadol (50mg in AM, 50mg in PM)
Haldol

😊 **What Makes Her Happy**
Visits from her son.

😣 **What Upsets Her**
When she feels lost and disoriented

Caregiver Interventions
Calming language
"Let's call your son"
Touching
Music

Rachel

Activities	Sleep 7 PM	Wake-Up	Breakfast	Morning Activities	Lunch	After Lunch Activities	Afternoon Nap	Dinner 5-6 PM	Turndown Time 6-7 PM
Action(s)		• Bangs on railing.<br>• Gets agitated when caregiver approaches.<br>• Gets cleaned<br>• Wheels to the dining room.	• Eats.<br>• Given meds.<br>• Wheels to the TV room.	• Watches TV with other residents in the living room for 45-60 minutes.<br>• Wheeled back to her room after watching TV.	• Wheeled to the lunch room.<br>• Eats softer foods.	• Son visits her daily.<br>• Watches TV in living room.<br>• Pet therapy<br>• Music therapy	• Gets changed.<br>• Eats snacks.	• Wears dentures.<br>• Eats softer foods.	
Health Condition							Severe Sundown Syndrome	Severe Sundown Syndrome	Severe Sundown Syndrome
Emotional State	😁	😡<br>Lonely	😑 😡	😑 😡	😑 😡	😁 😑<br>Calm	😡 😁	😑 😡	😑 😡
Caregiver Intervention		• Caregiver wakes, cleans, dresses, and performs ADL activities with her.<br>• Caregivers deals with Helen being upset.	• Caregiver wheels her to the dinner table.<br>• Caregiver spoon feeds her.	Caregiver wheels her to a chair/couch.	• Caregiver wakes, cleans, dresses, and performs ADL activities with her.<br>• Caregivers deals with Helen being upset.			• Caregiver gives medication to help her calm down.<br>• Caregiver wakes, cleans, dresses, and performs ADL activities with her.<br>• Caregivers deals with Helen being upset.	Caregiver cleans and prepares her for bed.
Touchpoint: Device				Uses iPad that is set up by caregiver.					
Opportunities for Robot-Human Interaction			Plays music to calm her down.	• Robot tells stories.<br>• Robot detects emotions.<br>• Robot shows pictures of family.					

Wanda

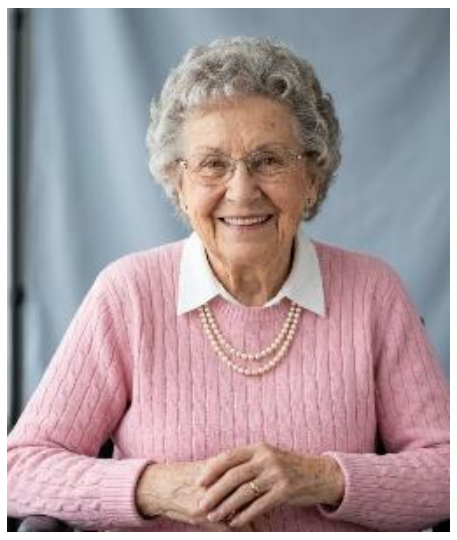

Overview
89-year old woman with severe dementia. She requires a wheelchair and cannot walk on her own.***She is often extremely upset.***

Level of Dementia
Severe

Health Condition
Overall generally healthy
Sleep quality generally good
Blood pressure: average
Chronic Diseases
Osteoporosis
Hearing
Good
Eyesight
Cataracts

Mobility
Wheelchair

Conversational Ability
Divorced from reality - she can speak clearly but her conversation does not address current reality. For example, when you ask her how she is doing, she asks why you ask.

She cries when a caregiver changes her, transfers her to wheelchair or couch. After a short period she adapts and stops crying.
Entertainment & Communication Devices Used
TV

Behaviors to Watch For
Caregivers need to give her space to express her emotions. Outbursts typically happen when a caregiver changes her, transfers her to wheelchair or couch.

Personality
Normally sedate and confused but becomes extremely emotional when there is any change in caregiver-provided activity (e.g. cleaning, transferring, feeding).

Interests
Only wants to be left alone.

Sleep Quality
Sleeps throughout the night.

Medications
Risperdal
Serouquel

😊 **What Makes Her Happy**
Sweet things like milkshakes, ice cream
Family visits

😞 **What Upsets Her**
Any change in activity (e.g. cleaning, transferring, feeding). Sometimes even asking her how she is doing is threatening to her

Caregiver Interventions
Allowing her to carry on with tantrum and reassuring her that she is safe.
Calming language
Touching

Wanda

Activities	Sleep 7 PM	Wake-Up	Breakfast	Morning Activities	Lunch	After Lunch Activities	Afternoon Nap	Dinner 5-6 PM	Turndown Time 6-7 PM
Action(s)	Goes to bed.	• Bed bath.<br>• Needs assistance to put on clothes.<br>• Wheels to the dining room.	• Eats.<br>• Given meds.<br>• Wheels to the TV room.	• Watches TV.<br>• Does puzzles.<br>• Colors painting books.	• Eat puree foods (lots of sweets for dessert) .<br>• Given meds (may crush in food).<br>• Wheels to the TV room.	• Sits in the Living Room for at least 30 minutes.<br>• Watches TV.<br>• Colors painting books.<br>• Does Puzzles and Bingo.<br>• Receives Physical therapy. (Given pain meds 30 min prior.)<br>• Participates in Music therapy/Pet therapy (alternating).<br>• Bed bath.<br>• Be visited by a healthcare provider (RN).	Takes a nap. (1-2 hrs.)	• Eats.<br>• Be given meds.	Gets ready for bed.
Health Condition	Sleep score: 76/100	Severe dementia	Poor short term memory		Cannot chew				
Emotional State	😁	😡	😭 😑 😡	😑 😡	😑 😡	😁 😑	😭 😡 😁	😑 😡	😭 😑 😡
Caregiver Intervention		• Caregiver wakes, cleans, dresses, and performs ADL activities with her.<br>• Caregiver helps to reorient her and calm her.	• Caregiver wheels her to the dinner table.<br>• Caregiver spoon feeds her.	Caregiver wheels her to a chair/couch.	• Caregiver wheels her to the dinner table.<br>• Caregiver spoon feeds her.		Caregiver cleans her incontinence.	• Caregiver wheels her to the dinner table.<br>• Caregiver spoon feeds her.<br>• Caregiver assesses whether she has Sundowning Syndrome and begins to get agitated.	Caregiver cleans and prepares her for bed.
Device Touchpoint	Pressure pad	Call button available (she forgets how to use it)							
Opportunities for Robot-Human Interaction	Constant supervision from robot		Once the robot senses she is crying, the robot comforts her and tells stories.	Robot makes sure she is being turned.			Robot alerts caregivers if there is wetness.	Assess for agitation	

AI Image Attribution: AI Image Attribution: The image of persons with dementia included in this multimedia appendix were generated with AI using Google Gemini and do not depict real individuals.

Reference: Google. (2024). Gemini [AI image generation tool]. https://gemini.google.com
