# Supplementary material for: Evaluation of Personas and Daily Activity Maps to Inform Clinical Care and Socially Assistive Robot Training and Design for Persons With Dementia: Mixed Methods Study
Source: J Med Internet Res. 2026 Oct 28;28:e85651. doi: 10.2196/85651 (PMC13718279; doi:10.2196/85651)
Supplement: Supplementary file 2 [file jmir-v28-e85651-s002.pdf]

Multimedia Appendix 2. Modified Persona Perception Scale (PPS).

Please fill out the Persona and Daily Activity Map (DAM) questionnaires for [*pseudo-patient's name*].

		**Persona Questions [pseudo-patient's name]**				
**No.**	**Questions**	**Fully Disagree**	**Somewhat Disagree**	**Neutral**	**Somewhat Agree**	**Fully Agree**
		**Completeness**				
**1**	The Persona is detailed enough to make decisions about the customers it describes.	☐	☐	☐	☐	☐
**2**	The Persona seems complete.	☐	☐	☐	☐	☐
**3**	The Persona provides enough information to understand the person it describes.	☐	☐	☐	☐	☐
**4**	The Persona is not missing vital information.	☐	☐	☐	☐	☐
		**Willingness to Use**				
**1**	I would make use of this Persona in my role in working with this person.	☐	☐	☐	☐	☐
**2**	I can imagine ways to make use of this Persona information in my role in working with this person	☐	☐	☐	☐	☐
**3**	This Persona would improve my ability to make decisions about the customers it describes.	☐	☐	☐	☐	☐
		**Credibility**				
**1**	The Persona seems to reflect the activities of a real person.	☐	☐	☐	☐	☐
**2**	I have met people like this Persona	☐	☐	☐	☐	☐
**3**	The picture of the Persona looks authentic.	☐	☐	☐	☐	☐
		**Clarity**				
**1**	The Persona information is well presented.	☐	☐	☐	☐	☐
**2**	The text in the Persona is clear enough to read.	☐	☐	☐	☐	☐

**3**	The information in the Persona profile is easy to understand.	☐	☐	☐	☐	☐
	**Empathy**					
**1**	I feel like I understand this Persona.	☐	☐	☐	☐	☐
**2**	I feel strong ties to this persona.	☐	☐	☐	☐	☐
**3**	I can imagine a day in the life of this persona.	☐	☐	☐	☐	☐

Daily Activity Map (DAM) Questions [pseudo-patient's name]

No.	Questions	Fully Disagree	Somewhat Disagree	Neutral	Somewhat Agree	Fully Agree
	**Completeness**					
**1**	The DAM is detailed enough to make decisions about the customers it describes.	☐	☐	☐	☐	☐
**2**	The DAM seems complete.	☐	☐	☐	☐	☐
**3**	The DAM provides enough information to understand the activities of the person it describes.	☐	☐	☐	☐	☐
**4**	The DAM is not missing vital information.	☐	☐	☐	☐	☐
	**Willingness to Use**					
**1**	I would make use of this DAM in my role with the person	☐	☐	☐	☐	☐
**2**	I can imagine ways to make use of this DAM information in my role in working with this person	☐	☐	☐	☐	☐
**3**	This DAM would improve my ability to make decisions about the customers it describes.	☐	☐	☐	☐	☐
	**Credibility**					
**1**	The DAM seems to reflect the activities of a real person.	☐	☐	☐	☐	☐
**2**	I have met people whose DAM is similar to this.	☐	☐	☐	☐	☐
**3**	The DAM of this persona looks authentic.	☐	☐	☐	☐	☐

	Clarity					
**1**	The DAM information is well presented.	☐	☐	☐	☐	☐
**2**	The text in the DAM is clear enough to read.	☐	☐	☐	☐	☐
**3**	The information in the DAM profile is easy to understand.	☐	☐	☐	☐	☐
	**Empathy**					
**1**	I feel like I understand the DAM of this persona.	☐	☐	☐	☐	☐
**2**	I feel strong ties to this persona and their DAM.	☐	☐	☐	☐	☐
**3**	I can imagine a day in the life of this persona.	☐	☐	☐	☐	☐

Reference: Salminen J, Santos JM, Kwak H, An J, Jung S, Jansen BJ. Persona Perception Scale: Development and Exploratory Validation of an Instrument for Evaluating Individuals' Perceptions of Personas. International Journal of Human-Computer Studies 2020 Sept;141:102437. doi: 10.1016/j.ijhcs.2020.102437
