# Supplementary material for: Evaluation of Personas and Daily Activity Maps to Inform Clinical Care and Socially Assistive Robot Training and Design for Persons With Dementia: Mixed Methods Study
Source: J Med Internet Res. 2026 Oct 28;28:e85651. doi: 10.2196/85651 (PMC13718279; doi:10.2196/85651)
Supplement: Supplementary file 4 [file jmir-v28-e85651-s003.docx]

Multimedia Appendix 3. Interview Guide.

Evaluation of Personas and Daily Activity Maps

in Dementia Care and Socially Assistive Robot Design

1. **Overview and Background**

*Interviewer: Throughout the interview, please use the term “person with dementia” or, when appropriate, “loved one,” rather than “PWD.”*

This interview guide is designed to engage family and professional caregivers of PWD, as well as dementia clinicians, researchers, and social robot designers, in evaluating personas and their corresponding Daily Activity Maps (DAMs) as design tools. This approach is consistent with user-centered design, in which the characteristics, needs, preferences, and daily experiences of PWD are considered when designing the visual and conversational interactions of Socially Assistive Robots (SARs). Participants’ evaluations and qualitative feedback will help inform the development of SARs that may support caregivers and clinicians in caring for PWD and assist designers in training and refining SAR interactions.

Although this study represents a preliminary and exploratory step in the user-centered design of SARs for PWD, it aims to address an important clinical need. Falls are the leading cause of injury and injury-related death among adults aged 65 years and older, and PWDs are at increased risk, with a reported 12-month fall rate of 45.5% [1,2]. Agitation has also been associated with increased fall risk among PWD [3]. We therefore hypothesize that incorporating design tools such as personas and DAMs to inform the design of SARs for PWD may help support interactions that reduce the frequency or intensity of agitation episodes and, ultimately, may contribute to reducing agitation-related falls.

In this study, our SAR, Navi, uses an iPad-style screen to interact visually and conversationally with PWD (see Figure 1 below).

**Figure 1**. Navi, a socially assistive robot.

# 2. Methods

***** *Interviewer: Please use the term “Daily Activity Map” rather than “DAM” throughout the interview.*

Thank you again for participating in this study. Your opinions are important to us. Before we begin, we will define several terms used throughout this interview:

1. **User-centered design:** an approach to design that focuses on users and their needs throughout the design process [4].
2. **Persona:** a fictional, research-informed representation of a target user or user group. In this study, personas represent PWD and summarize relevant user characteristics, needs, behaviors, and care-related challenges [5].
3. **Daily Activity Map:** a visual representation of a person’s typical day that organizes activities and routines over time and may include relevant behaviors, experiences, needs, and emotions. In this study, DAMs depict the person’s activities from waking until bedtime, as well as care-related needs.

In preparation for this interview, our expert team developed nine pairs of personas and corresponding DAMs representing PWD. Names and other potentially identifying details have been changed to protect privacy. The images are AI-generated while preserving key characteristics of the PWD.

3. **Procedure**

We appreciate your participation in this research study. We expect each meeting to last approximately one hour and may need two to three meetings with you to accomplish the study goals. Prior to the interview, we asked you to review the nine personas and their corresponding DAMs and select up to three that you felt you could identify with or that resembled someone you know in some way. We believe that making this connection may help make your feedback more meaningful. Thank you for identifying your selected personas and DAMs.

During this meeting, to help inform the design and training of the SAR, we will review each selected persona and its corresponding DAM individually. For each persona-DAM pair, we will ask you a set of questions and record your responses. We will then ask follow-up questions to better understand why you selected each response.

** Interviewer: Please note that the survey and interview questions are intended to help address the following research questions:*

1. *How well do the current personas and DAMs describe the characteristics of PWD and their representative daily activities?*
2. *How well do these personas and DAMs identify potential concerns or situations that may trigger agitation or emotional disturbances requiring a care response or intervention?*
3. *Using these personas and DAMs, what potential opportunities are there for the SAR to prevent or respond to these triggers?*

4. **Questions**

**Persona Evaluation**

Please carefully review the persona of *[persona’s name]* presented below. Take as much time as you need to read through each card. If you have any questions, please ask them before we begin. I will read each question aloud and ask you to indicate your level of agreement on a scale from 1, “fully disagree,” to 5, “fully agree.” Following each section, I will ask you to explain why you selected your rating and what, if anything, could be improved.

** Interviewer: Please display the persona and the modified PPS scale on the screen and read each question aloud. Probe as needed to better understand the participant’s response or suggestions for improvement:*

- *For item #, would you mind telling us why you rated this persona this way?*
- *What do you think is missing, and why?*
- *Is there anything you would like to add?*

**Trigger Questions**

On a scale from 1, “fully disagree,” to 5, “fully agree,” how well does the persona realistically identify situations or events that may trigger agitation and require a care response from the SAR (e.g., personal storytelling)?

| 1 | 2 | 3 | 4 | 5 |
| --- | --- | --- | --- | --- |
| Fully Disagree | **Somewhat Disagree** | **Agree** | **Somewhat Agree** | **Fully Agree** |

- Please explain why you selected this response.
- For responses not scored as “Fully Agree,” how could the situation or event be better identified to improve your score?
- Where in the persona can these triggers be identified?

**Daily Activity Map Evaluation**

Next, we will focus on the DAM of *[persona’s name]*. Please read the DAM carefully and take as much time as you need. If you have any questions, please wait until we have completed the next set of closed-ended questions. I will read each question aloud and ask you to indicate your level of agreement on a scale from “Fully Disagree” to “Fully Agree.”

** Interviewer: Please display the DAM and the modified PPS scale on the screen and read each question aloud. Probe as needed to better understand the participant’s response or suggestions for improvement:*

- *For item #, would you mind telling us why you rated this persona this way?*
- *What do you think is missing, and why?*
- *Is there anything you would like to add?*

**Trigger Questions**

On a scale from 1, “Fully Disagree,” to 5, “Fully Agree,” how well does the DAM realistically identify situations or events that may trigger agitation and require a caring response from the SAR (e.g., personal storytelling)?

| 1 | 2 | 3 | 4 | 5 |
| --- | --- | --- | --- | --- |
| Fully Disagree | **Somewhat Disagree** | **Agree** | **Somewhat Agree** | **Fully Agree** |

- Please explain why you selected this response.
- For responses not scored as “Fully Agree,” how could the situation or event be better identified to improve your score?
- Where in the DAM can these triggers be identified?

**Opportunities for Interactive Interventions**

Focusing now on opportunities for designing interactive interventions between the SAR and a PWD, please answer the following questions as best you can.

1. How would you expand on or change the existing opportunities identified, based on your professional experience with PWD, and why?
2. What additional opportunities do you see throughout the day, and why? Please describe the trigger and the nature of the opportunity in your own words.
3. Would you mind spending a few minutes writing down or explaining a scenario for your envisioned opportunity? You may describe multiple scenarios.
4. What features of the SAR do you think might encourage a PWD to engage in conversation?

**FOR ENGINEERS**

What design requirements should be considered for the SAR user interface (e.g., font size, background color, or volume of the verbal response)?

5. **Final Wrap-up**

We have now completed the evaluation of one persona and its DAM. Is there anything else that comes to mind that you would like to add?

Your feedback has been very helpful. Would you like to schedule another meeting to discuss the second and third persona-DAM pairs?

We would like to thank you for your time and valuable contribution to this research.

**References:**

1. Kakara R, Bergen G, Burns E, Stevens M. Nonfatal and fatal falls among adults aged ≥65 years-United States, 2020-2021. MMWR Morb Mortal Wkly Rep. 2023;72:938-943. doi:10.15585/mmwr.mm7235a1.
2. Okoye SM, Fabius CD, Reider L, Wolff JL. Predictors of falls in older adults with and without dementia. Alzheimers Dement. 2023;19(7):2888-2897. doi:10.1002/alz.12916.
3. Fillit H, Aigbogun MS, Gagnon-Sanschagrin P, Cloutier M, Davidson M, Serra E, Guérin A, Baker RA, Houle CR, Grossberg G. Impact of agitation in long-term care residents with dementia in the United States. Int J Geriatr Psychiatry. 2021;36(12):1959-1969. doi:10.1002/gps.5604.
4. De Vito Dabbs A, Myers BA, Mc Curry KR, Dunbar-Jacob J, Hawkins RP, Begey A, Dew MA. User-centered design and interactive health technologies for patients. Comput Inform Nurs. 2009;27(3):175-183. doi:10.1097/NCN.0b013e31819f7c7c.
5. Miaskiewicz T, Kozar KA. Personas and user-centered design: how can personas benefit product design processes? Des Stud. 2011;32(5):417-430. doi:10.1016/j.destud.2011.03.003.
